# Porcine Expanded Potential Stem Cells as a Versatile Platform for Multiplex Genome Editing and Immunophenotyping in Xenotransplantation

**DOI:** 10.1111/xen.70161

**Published:** 2026-08-21

**Authors:** Yiyi Xuan, Yong Xiang, Xining Wang, Björn Petersen, Pentao Liu

**Affiliations:** ^1^ InnoHK Centre for Translational Stem Cell Biology Hong Kong Science Park, Hong Kong Special Administrative Region China; ^2^ School of Biomedical Sciences, Li Ka Shing Faculty of Medicine The University of Hong Kong Hong Kong, Hong Kong Special Administrative Region China; ^3^ School of Biomedical Sciences The Chinese University of Hong Kong Shatin, Hong Kong Special Administrative Region China; ^4^ FLI, Institute of Farm Animal Genetics Mariensee Germany

**Keywords:** genome editing, immunophenotyping, porcine stem cells, xenotransplantation

## Abstract

Xenotransplantation utilizing pig donors offers a promising solution to organ shortages, but immune incompatibilities across species remain a major challenge. Current reliance on porcine primary fibroblasts for genome editing is limited by low inefficiency in complex gene editing and difficulties in immunophenotyping edited cells. In this study, we demonstrate that porcine expanded potential stem cells (pEPSCs), derived from preimplantation embryos, provide a robust and versatile platform for generating donor cells for xenotransplantation. These pluripotent cells can differentiate into both embryonic and extraembryonic lineages, maintain genetic stability through multiple edits, and enable precise genome modifications. We performed multiple gene knockouts in pEPSCs targeting key immune rejection genes and precisely inserted a genetic cassette to facilitate streamlined introduction of human cDNAs via cassette exchange. The engineered pEPSCs retained their pluripotency and stability even after multiple rounds of genome editing. When differentiated into endothelial cells, they exhibited high immunogenicity, serving as a rapid and quantitative platform for immune response assessment. Importantly, the edited endothelial cells exhibited substantially reduced immunogenicity, confirming the functional impact of the genetic modifications. Although we have not yet generated live pigs from these gene‐edited pEPSCs via somatic cell nuclear transfer (SCNT), our findings establish pEPSCs as a novel and improved platform for generating genetically engineered pig donors and for functionally evaluating genetic modifications, thereby advancing the prospects of xenotransplantation.

## 1 Significance Statement

Porcine expanded potential stem cells (pEPSCs) offer a transformative tool for advancing xenotransplantation. Their capacity for highly efficient genetic modification allows us to precisely edit pig genomes to make organs more compatible for human transplantation. Furthermore, these cells can be differentiated into various cell types, providing a critical cell resource for studying the human immune response to pig tissues in vitro. This research bridges a crucial gap, enabling both the creation of improved donor pigs and a deeper understanding of transplant immunology, therefore accelerating the development of safe and effective clinical xenotransplantation.

## 2 Main Text

### 2.1 Introduction

The shortage of transplantable organs remains a critical global challenge. In the United States alone, over 103 000 individuals await organ transplants, with an alarming average of 13 lives lost daily due to scarcity of suitable organs (HRSA, 2026) [1]. This urgent need has driven research toward innovative solutions, particularly xenotransplantation—the use of animal organs, especially from pigs, as donors. Pigs are considered ideal donors due to their physiological similarities to humans in anatomy, function, metabolism, and genetics, coupled with their relatively low maintenance costs [2].

Recent progress in xenotransplantation has yielded promising results. For example, pig‐to‐human organ transplants have demonstrated survival periods approaching two months [3, 4, 5, 6, 7, 8, 9, 10]. Even more impressive are cases where pig‐to‐non‐human primate (NHP) transplants have survival up to 758 days [11]. Despite these advances, clinical outcomes remain suboptimal, underscoring the need for further improvements in organ survival and functionality, and strategies to overcome cross‐species immune incompatibilities [11, 12, 13, 14, 15, 16, 17, 18, 19].

Traditionally, pigs for xenotransplantation are generated via somatic cell nuclear transfer (SCNT) (Figure S1A) [3, 11, 20, 21]. The donor cells used are typically fibroblasts, which are genetically modified to inactivate genes implicated in immune rejection and organ acceptance and to express some human cDNAs for regulating coagulation, modulating complement activation, and improving graft survival. The inactivated genes include those encoding for key saccharide antigens responsible for severe hyperacute rejection and for rapid organ failure, such as *GGTA1* for α‐Gal (galactose‐α‐1,3‐galactose) epitope synthesis [22, 23], *CMAH* for Neu5Gc (N‐glycolylneuraminic acid) [22, 24], and *B4GALNT2* for Sd (a) (Sia‐α2.3‐[GalNAc‐β1.4]Gal‐β1.4‐GlcNAc) [25]. On the other hand, human immune‐modulating genes like *CD46* [3, 11, 21, 26, 27, 28, 29]*, CD47*[3, 11, 21, 30, 31], *THBM* [3, 11, 21, 26, 27, 29, 32], *HO‐1* [3, 11, 33, 34], *A20* [11, 20, 35, 36], *HLA‐E* [21, 37, 38, 39], HLA‐G [37, 40] have been transgenically expressed to improve compatibility. Additionally, knockout of growth hormone receptor gene (*GHR*) helps prevent excessive organ growth, enabling xenograft survival for up to nine months in NHP models [41].

However, several technical hurdles still hinder the efficient production of genetically modified pigs. For instance, efforts are underway to gain deeper insights into the somatic cell epigenome reprogramming process and to reduce developmental anomalies and enhance cloning efficiency [42, 43, 44, 45]. Another challenge is improving genome‐editing in porcine fibroblasts, which have limited self‐renewal capacity, making complex genome‐editing challenging [46, 47]. As a result, generating pigs with multiple genetic modifications often requires multiple rounds of SCNT, a process that is labor‐intensive, time‐consuming, and resource‐demanding [45, 48]. Although DNA transposition offers a straightforward and efficient method for integrating transgenes into the porcine genome [21, 49], it lacks precision control over copy number and insertion site [50], leading to variability in transgene expression. A particularly important issue is that fibroblasts express only a limited set of xeno‐antigens relevant to transplantation, making them unsuitable for directly evaluating immune compatibility immediate after the editing. Consequently, assessing the functional effects on xenotransplantation, especially immunocompatibility, of a given genetic modification requires testing through cloned embryos or live animals. Since endothelial cells, particularly tissue‐specific endothelial cells, express high and physiologically relevant xeno‐antigens, they are commonly used for immunocompatibility evaluations [11, 21]. Ideally, genome editing should be performed in stem cells, allowing for direct assessment of the functional consequence.

pEPSCs are established from pre‐implantation embryos of various genetic backgrounds, including SCNT embryos, or by reprogramming somatic cells [51, 52]. These cells are pluripotent, capable of long‐term culture, and maintain genetic and epigenetic stability [51, 52]. Importantly, we have demonstrated that pEPSCs enable precision and efficient genome‐editing [52]. These unique properties of pEPSCs make them a promising platform for advancing xenotransplantation research. Building on these advantages, we generated pEPSCs with multiple targeted genetic modifications. Specifically, we inactivated *GGTA1*, *CMAH*, *B4GALNT2*, and *GHR* to address immune rejection and abnormal growth issues. Additionally, we inserted a recombinase‐mediated cassette exchange (RMCE) cassette into the *ROSA26* locus, enabling efficient and precise transgene integration of the human CD47 cDNA into the porcine genome [53, 54].

The genetically modified pEPSCs retained pluripotency and genetic stability even after multiple rounds of editing. Their ability to differentiate into various cell types enabled the generation of highly immunogenic endothelial cells for rapid, quantitative assessment of immune responses in vitro. The pEPSC approach thus leverages the precise gene‐editing capacity of pEPSCs combined with streamlined immunophenotyping, potentially accelerating the development of suitable pig donors for xenotransplantation. At this moment, the gene‐edited pEPSCs are being evaluated in SCNT for the production of live pigs.

## 3 Results

### 3.1 Genome Editing in Porcine EPSCs for Xenotransplantation

**FIGURE 1:**
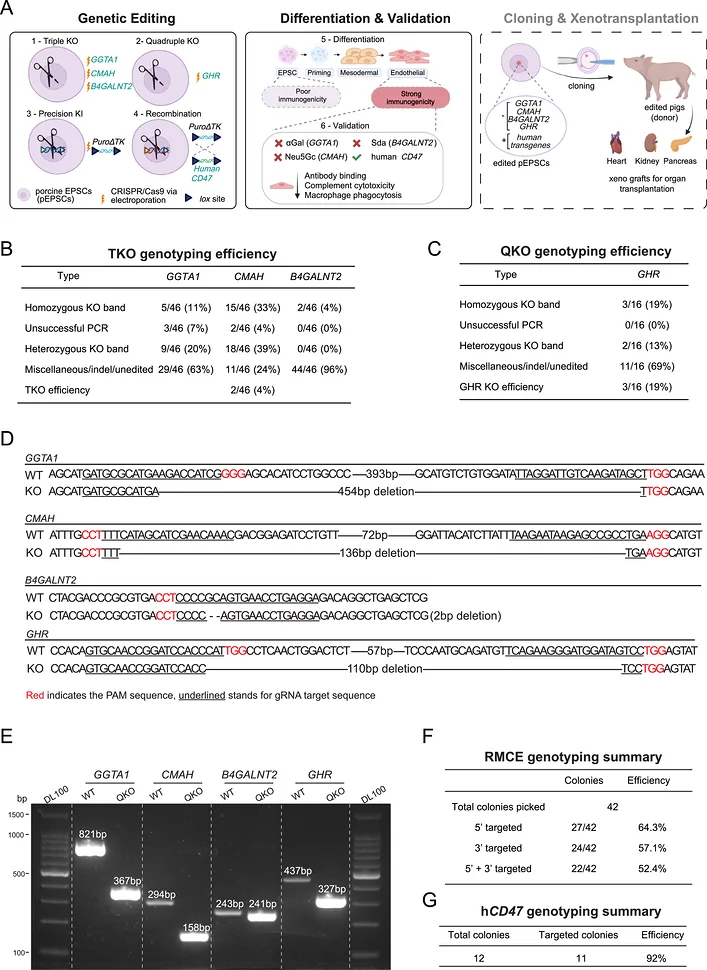
Serial genome editing in porcine EPSCs (pEPSCs) for xenotransplantation. (A) Working model shows performing multiple rounds of genome editing in pEPSCs and the subsequent in vitro immunophenotyping using pEPSC‐derived endothelial cells for assessing immunocompatibility. This approach supports the future generation of immune‐compatible porcine organs for clinical transplantation. (B) Genotyping summary of generation of triple knockout (TKO) pEPSCs. (C) Genotyping summary of *GHR* KO efficiency. (D) Sanger sequencing confirmation of out‐of‐frame genomic deletions in quadruple KO (QKO) pEPSCs targeting four genes (*GGTA1/CMAH/B4GALNT2/GHR*). (E) Agarose gel electrophoresis image displays the genotyping results for QKO‐pEPSCs. Visible PCR fragment shifts are observed for *GGTA1*, *CMAH*, and *GHR*. The *B4GALNT2* gene, with a 2‐base pair deletion, did not exhibit an obvious band shift. (F) Genotyping summary of recombinase mediated cassette exchange (RMCE) cell line construction, revealing the *lox*66‐puroΔTK‐*lox*2272 cassette knock in (KI) efficiency at the *ROSA26* locus. (G) Genotyping for human *CD47 (hCD47)* integration events at the *ROSA26* locus by RMCE.

We took advantage of the culture robustness and efficient precision genome editing of pEPSCs and performed multiple rounds of editing with the goal of obtaining donor cells suitable for xenotransplantation (Figure 1A). We first sought to generate a triple knockout (TKO) pEPSC by inactivating *GGTA1* (α‐Gal), *CMAH* (Neu5Gc), and *B4GALNT2* (Sd(a)) in a single transfection event using the CRISPR/Cas9.

To achieve this, we designed pairs of sgRNAs (two per gene, total six sgRNAs; Table S1) targeting specific exons of each gene to induce large deletions. For *GGTA1*, we targeted exon 7 with two sgRNAs to induce a large deletion encoding C‐terminal catalytic domain, thus eliminating α‐Gal epitope expression (Figure S1). Similarly, for *CMAH*, two sgRNAs targeted exon 1 to induce a large deletion disrupting its hydroxylase function and Neu5Gc synthesis; for *B4GALNT2*, two sgRNAs targeted exon 3 to induce a large deletion impairing its galactosaminyl transferase function and disrupting Sd(a) epitope expression.

All six sgRNAs, together with Cas9 protein, were co‐delivered into pEPSCs by electroporation in a single step. The transfected cells were subsequently cultured to allow colony formation for genotyping (Table S2) [52]. Genomic DNA PCR genotyping revealed that 11% of the colonies were homozygous knockout mutants for *GGTA1* and 20% displayed the heterozygous deletion band (Figure 1B, Figure S2). For deleting exon 1 of the *CMAH* locus, we obtained 33% homozygous deletion mutants and 39% heterozygous colonies. However, targeting *B4GALNT2* yielded a significantly lower editing efficiency, with only 4% showing a 2 bp deletion and 96% displaying miscellaneous indel or unedited patterns. This reduced editing efficiency is likely due to its conserved paralog *B4GALNT2‐like* (*LOC110255214*) [55]. Both *B4GALNT2* and *B4GALNT2‐like* encode functionally similar β‐1,4‐N‐acetylgalactosaminyltransferases, with *B4GALNT2‐like* retaining 58.7% of the enzymatic activity of *B4GALNT2* [55]. Notably, our editing attempts successfully inactivated both the *B4GALNT2* and *B4GALNT2‐like* (*LOC110255214*) loci (Figure S3A, S3B). Despite these genes being located in close proximity on chromosome 12, PCR analysis confirmed that intervening genes (*LOC110255205, LOC102158609*, *LOC110255211, and TRANQ‐UUG*) remained unaffected by the editing (Figure S3C, Table S2). This functional compensation between paralogs likely accounts for the observed editing challenges. Consequently, among the 46 colonies genotyped, the overall efficiency of achieving the TKO was 4% (Figure 1B).

To address the potential porcine organ overgrowth in transplant recipients [56, 57], we inactivated the *GHR* gene in the TKO pEPSCs for the quadruple knockout (QKO) pEPSCs. The dual‐sgRNA system targeting exon 7 of *GHR* was designed to generate a ∼110 bp large deletion, thereby disrupting the extracellular domain of the protein (Figure S1). PCR genotyping identified 19% colonies exhibited clear homozygous deletions at the *GHR* locus (Figure 1C, Figure S2D). The genetic perturbation information and genotyping details for generating the QKO‐pEPSCs are summarized in Figures 1D, 1E, S1 and S2.

We next explored using CRISPR/Cas9 mediated knock‐in followed by recombinase‐mediated cassette exchange (RMCE) to precisely engineer human cDNAs into the porcine genome [53]. We first established a master pEPSC line where the RMCE cassette carrying two distinct *lox* sites for recombination was inserted into the *ROSA26* locus. Human cDNAs or other transgenes can be conveniently introduced into the locus in a two‐step approach (Figure 1A, Figure S4A). In the first step, the *puroΔTK* cassette containing the CAG promoter flanked by *lox66* and *lox2272* sites was targeted to the *ROSA26* locus via CRISPR/Cas9‐mediated homology‐directed repair in the QKO‐EPSCs (Figure 1A, Figure S4A). The correctly targeted colonies were first selected out by puromycin and identified by genomic DNA PCR detecting the junction fragments and confirmed by Sanger sequencing (Figure 1F, Figure S4B, S5). Among the 42 colonies analyzed, 22 were confirmed to be correctly targeted, representing a targeting efficiency of 52.4% (Figure 1F).

In the QKO‐RMCE master pEPSCs, we next replaced the *puroΔTK* cassette with the gene of interest (GOI) via Cre‐mediated cassette exchange (Figure 1A, Figure S4A). As a proof‐of‐principle, we tested introducing human *CD47* cDNA into the *ROSA26* locus (Figure S4A). Human *CD47* cDNA was flanked by *lox71* and *lox2272* sites at its ends, ensuring that recombination between *lox71* and *lox66* generated a mutant non‐functional *lox* site to minimize the potential recycling between the donor cassette and the recipient locus, ensuring high cassette exchange efficiency (Figure S4A). To further improve correct integration efficiency, we applied negative selection using Ganciclovir to eliminate cells where the *puroΔTK* cassette was retained due to incomplete Cre‐*lox* recombination. After Cre site‐specific recombination and Ganciclovir negative selection, the CAG promoter‐driven *hCD47* was successfully integrated into the *ROSA26* locus. Junction PCR genotyping and Sanger sequencing confirmed that 11 out of 12 colonies (92%) carried the desired integration (Figure 1G, Figure S6). In summary, pEPSCs enabled the construction of porcine stem cells carrying multiple rounds of genome editing including both porcine xeno‐gene knockouts and human immune‐modulating transgene precision integration.

### 3.2 Molecular and Developmental Characterization of Q47‐EPSCs

**FIGURE 2:**
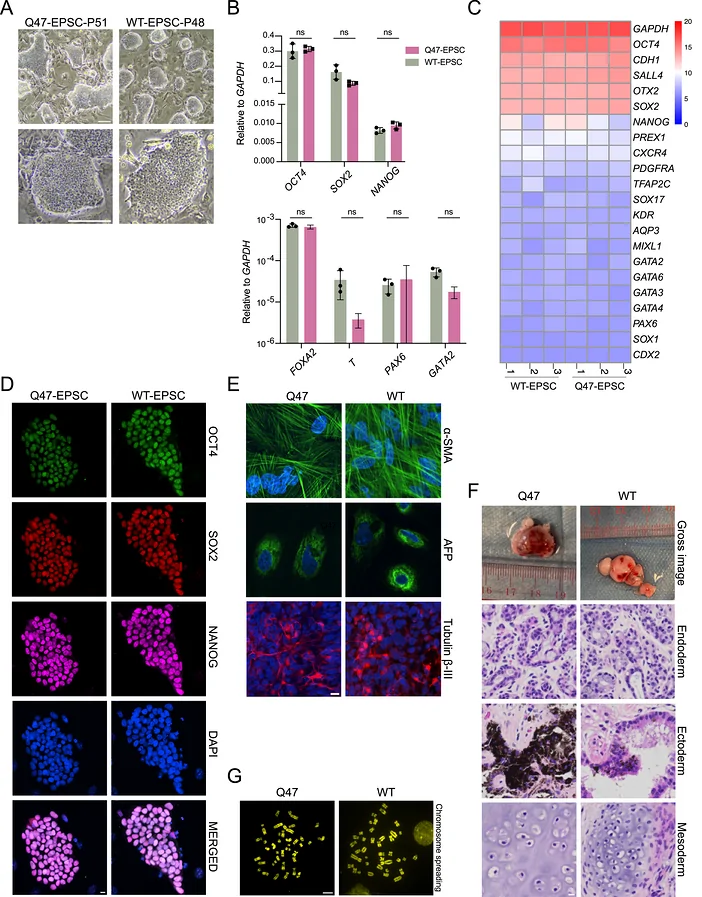
Molecular characterization of genome‐edited pEPSCs. (A) Bright‐field images of normal or wild‐type (WT) pEPSCs at passage 48 (WT‐pEPSC‐P48), and Q47‐pEPSCs at passage 51 (Q47‐pEPSC‐P51). Scale bars: 100 µm. (B) Expression of pluripotency genes (*OCT4*, *SOX2*, *NANOG*) and lineage genes (endoderm: *FOXA2*; mesoderm: *T*; ectoderm: *PAX6*; extra‐embryonic: *GATA2*) by qRT‐PCR in WT‐pEPSCs and Q47‐pEPSCs. Error bars indicate ± SD (N = 3, independent experiments). ns, *P* ≥ 0.05. Statistical analysis was performed using unpaired Mann–Whitney test. (C) Transcriptional profiles of pluripotency and lineage markers in WT and Q47 pEPSCs in bulk RNA sequencing analysis. (D) Immunofluorescence staining of pluripotency factors (POU5F1, SOX2, NANOG) in WT‐pEPSCs and Q47‐pEPSCs. (E) In vitro embryoid body formation assay of WT‐pEPSCs and Q47‐pEPSCs. Immunofluorescence staining detection of positive staining of AFP (endoderm), α‐SMA (mesoderm), and tubulin β‐III (ectoderm). (F) Differentiation of WT‐pEPSCs and Q47‐pEPSCs in teratoma formation assay. (G) Karyotyping of WT‐pEPSCs and Q47‐pEPSCs. Scale bars: 5 µm. (D & E) Nuclei were counterstained with DAPI (blue). *N* = 3, independent experiments. (D, E & G) Scale bars: 10 µm.

To evaluate Q47‐EPSCs (referred to as QKO pEPSCs with human CD47 integration), we compared them with the unedited EPSCs (WT‐EPSCs). Q47‐EPSCs exhibited robust proliferation (Figure S7A, S7B), forming colonies with a morphology similar to WT‐EPSCs—domed‐shaped with a light periphery and densely packed cells (Figure 2A). Transcriptomic profiling exhibited high similarity between Q47‐EPSCs and WT‐EPSCs (Figure S7C), further supported by both heatmap visualization of global gene expression patterns and MA plot analysis, which showed symmetric distribution of most genes around the zero baseline (indicating no fold‐change difference), with only a limited number of differentially expressed genes exhibiting significant variation (Figure S7D, S7E), indicating that extensive genetic editing did not substantially affect overall transcriptional profiles.

Specifically, molecular analysis by qPCR and bulk RNA sequencing demonstrated that Q47‐EPSCs expressed comparable high levels of key pluripotency genes *OCT4*, *SOX2*, *NANOG* and *SALL4*, similar to WT‐EPSCs (Figures 2B, 2C, Table S3). Conversely, the expression of lineage‐specific genes such as *FOXA2*, *SOX17*, *GATA6*, *CXCR4* (endoderm); *T, MIXL1, GATA4, KDR* (mesoderm); *PAX6, SOX1, NESTIN, TUBB3* (ectoderm); and *GATA2, GATA3, TFAP2C, CDX2, AQP3* (trophectoderm and amnion) remained low or undetectable, consistent with an undifferentiated pluripotency state (Figures 2B, 2C). Immunofluorescence staining further validated expression of the pluripotency factors (Figure 2D, Table S4).

We next assessed their developmental potential by performing both in vitro and in vivo differentiation assays. In vitro differentiation of Q47‐EPSCs in embryoid body (EB) assay generated derivatives of all the three germ layers, as evidenced by neural (ectoderm), muscle (mesoderm), and AFP‐positive (endoderm) cell populations (Figure 2E). In the teratomas formed from Q47‐EPSCs in immunocompromised mice, histological analysis also confirmed the presence of cells derived from all the three somatic germ layers (Figure 2F), demonstrating their pluripotency potential. Karyotyping analysis revealed that porcine Q47‐EPSCs maintained a normal diploid chromosome count (Figure 2G, Table S5), indicating that the genome remained stable despite multiple rounds of genome editing. To further assess the genomic safety of the multiplex genome editing, we performed genome‐wide off‐target prediction followed by whole‐genome sequencing (WGS) validation for all 9 sgRNAs. A total of 913 potential off‐target sites were predicted (allowing up to 3 mismatches). WGS analysis of Q47‐pEPSCs revealed no insertion/deletion (INDEL) or structural variant (SV) off‐target candidates within ± 10 bp of any predicted site (Table S6). These data confirm that the multiplex genome editing did not introduce detectable off‐target mutations in the Q47‐pEPSC line. The fact that Q47‐EPSCs, despite undergoing quadruple gene knockouts and human *CD47* integration, maintain their pluripotency, developmental potential and genome integrity highlights the promising potential of pEPSCs as a valuable donor cell source for advancing xenotransplantation research.

### 3.3 Q47‐PEPSCs Express Minimal Levels of Xeno‐Antigens and Are Not Suitable for Immunophenotyping

Besides culture robustness and efficient precision gene editing, a major advantage of pEPSCs is their ability to differentiate into various cell types, potentially enabling immunophenotyping without having to go through SCNT embryos.

**FIGURE 3:**
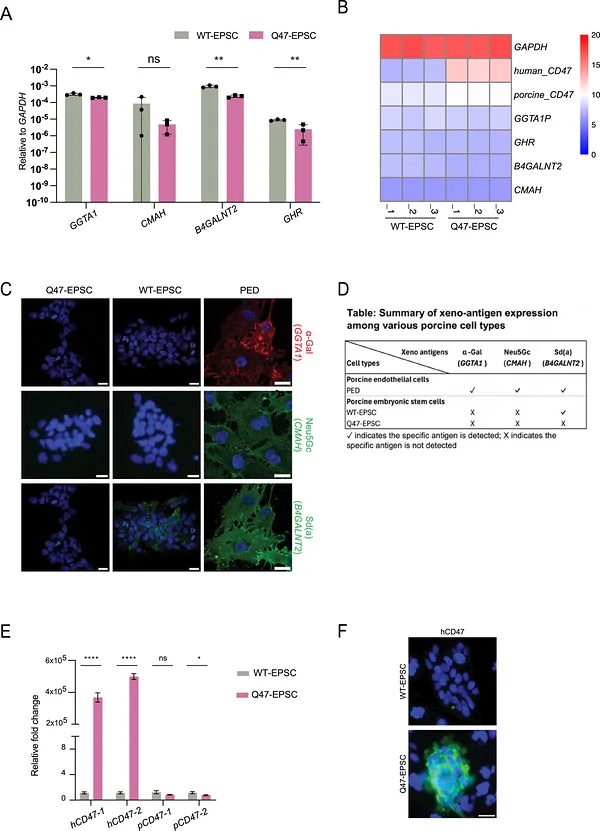
Validation of genome editing and gene expression in pEPSCs. (A) RT‐qPCR analysis of *GGTA1*, *CMAH*, *B4GALNT2*, and *GHR* in WT‐pEPSCs and Q47‐pEPSCs. (B) Bulk RNA‐Seq analysis of xeno‐related genes and *CD47* (human and porcine) in WT‐pEPSCs and Q47‐pEPSCs. (C) Immunofluorescence staining showing expression of α‐Gal (*GGTA1*), Neu5Gc (*CMAH*), and Sd(a) (*B4GALNT2*) in WT‐pEPSCs, Q47‐pEPSCs and porcine endothelial cell line PED‐SV15 (hereafter referred to as PED) Nuclei were counterstained with DAPI (blue). Scale bars: 20 µm. (D) Summary of xeno‐antigens on PED, WT‐pEPSCs and Q47‐pEPSCs. (E) RT‐qPCR analysis of human and porcine *CD47* expression in WT‐pEPSCs and Q47‐pEPSCs. (F) Immunofluorescence staining of human CD47 in WT‐pESPCs and Q47‐pEPSCs. Nuclei were counterstained with DAPI (blue). Scale bars: 25 µm. (A, E) Error bars indicate ± SD (*N* = 3, independent experiments). ns, *P* ≥ 0.05. *P < 0.05, **P < 0.0,1 ****P < 0.0001. Statistical analysis was performed using unpaired Student's *t*‐test.

To evaluate the functional consequence of genome editing in pEPSCs, we examined the edited porcine genes and the integrated human *CD47* cDNA in WT‐pEPSCs and Q47‐pEPSCs. However, in WT‐pEPSCs, the transcriptional levels of *GGTA1*, *CMAH*, *B4GALNT2*, and *GHR* were nearly undetectable (Figures 3A, 3B). Consistent with low gene expression, immunofluorescence staining did not detect the abundant presence of the antigenic epitopes like α‐Gal (*GGTA1*), Neu5Gc (CMAH) in either WT‐pEPSCs or Q47‐pEPSCs (Figure 3C). This was further supported by flow cytometry analysis (Figure S8A). Notably, α‐Gal and Neu5Gc were not detected in both lines, whereas Sd(a) was only minimally present in WT‐pEPSCs. As a result, undifferentiated pEPSCs exhibited only weak binding to pre‐formed human IgG and IgM antibodies (Figure S8B, S8C), comparable to human embryonic stem cells (hESCs), which served as a negative control (Figure S8C). Consistently, human complement‐mediated cytotoxicity assay showed that undifferentiated pEPSCs, regardless of genotype (WT or Q47), exhibited low specific cytotoxicity comparable to hESCs (Figure S8D, S8E).

In contrast to hypoimmunogenic pEPSCs, the immortalized porcine endothelial cell line PED‐SV15 (hereafter referred to as PED) strongly expressed the xeno‐antigens (Figures 3C, 3D). Therefore, undifferentiated pEPSCs are not suitable for direct immunophenotypic analysis of xeno‐antigens due to very low or undetectable expression of these epitopes. pEPSCs need to differentiate to cell types such as highly immunogenic endothelial cells, in order to reliably assess the functional outcome of the gene modifications.

In addition to xeno‐antigens, we examined also *CD47* expression in undifferentiated pEPSCs. Both WT‐pEPSCs and Q47‐pEPSCs expressed negligible levels of the endogenous *pCD47* (Figures 3B, 3E). In contrast, Q47‐pEPSCs expressed high levels of human *CD47* (*hCD47*) detected by RT‐qPCR (Figures 3B, 3E) and by immunostaining (Figure 3F), validating transgenic CD47 expression.

### 3.4 Efficient Generation of Endothelial Cells From pEPSCs

Endothelial cells play a pivotal role in the success of porcine‐to‐human xenotransplantation. They form the inner lining of blood vessels, ensuring their structural integrity and stability. Endothelial cells express porcine Major Histocompatibility Complex (MHC) antigens [58, 59] and xeno‐antigen epitopes such as α‐Gal, Neu5Gc, and Sd(a). These antigens can trigger an immune response, leading to graft destruction [60]. Additionally, endothelial cells regulate coagulation by secreting factors like von Willebrand factor and tissue factor pathway inhibitor, which balance clot formation and prevention [26]. Therefore, the compatibility of donor pig endothelial cells with the human immune system is crucial for graft survival and function and for preventing complications such as clotting, thrombosis, and immune rejection [26].

As discussed, a major advantage of porcine EPSCs to serve as a novel cell source for xenotransplantation lies in their ability to differentiate into all cell types [51, 52], particularly in immunophenotyping. Given that PED showed high xeno‐relevant immunogenicity (Figures 3C, 3D), we differentiated WT‐ and Q47‐pEPSCs into endothelial cells to quantitatively assess xeno‐antigen expression and evaluated the compatibility with the human immune system.

**FIGURE 4:**
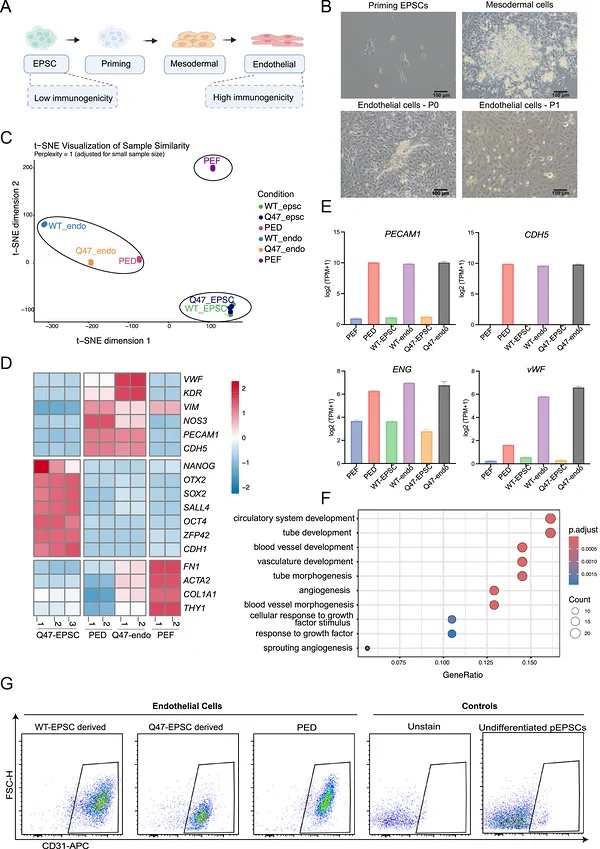
Generation of endothelial cells from pEPSCs. (A) Schematic view of the endothelial generation protocol from pEPSCs, which are primed, guided towards mesoderm and eventually committed to endothelial cells, from possessing low to high immunogenicity. (B) Brightfield images showing morphological changes during endothelial generation. Scale bars: 100 µm. (C) t‐SNE projection clustering pEPSC‐derived endothelial cells (WT‐endo, Q47‐endo) with PED as the control, but distinct from porcine embryonic fibroblasts (PEF) and undifferentiated pEPSCs. (D) Heatmap of differentially expressed genes across clusters (endothelial, fibroblast, pEPSC). (E) RNAseq analysis detection of expression of endothelial marker genes (*PECAM1*, *CDH5*, *vWF*, and *ENG*) in WT‐endo and Q47‐endo cells, compared to pEPSCs, PED and PEF. (F) Gene ontology (GO) enrichment analysis of highly variable genes in pEPSC‐derived endothelial cells. (G) Flow cytometry detection of CD31 on endothelial cells derived from WT‐pEPSCs, Q47‐pEPSCs, or PED, as well as undifferentiated pEPSCs and unstained controls. Experiments were repeated twice with similar results. Data in (C, D, E & F) were from bulk RNA‐seq.

We modified a published three‐step protocol for generating endothelial cells from human ESCs [61], and successfully generated endothelial cells from pEPSCs (Figures 4A, 4B and S9A). This process began with culturing pEPSCs in Essential 8 medium [62], which mimics the post‐implantation environment and primes the cells for differentiation (Figures 4A, 4B, S9A). Next, a mesoderm‐induction medium was applied for two days to guide commitment to mesoderm lineages. Finally, early endothelial progenitors were produced using an endothelial cell induction medium. Hemogenic endothelial cell aggregates (Figure 4B, S9A), indicative of hematopoietic potential, were observed after one day of endothelial medium induction. Between days 7 and 9 of differentiation, endothelial cells were harvested for CD31 analysis or further passaging and expansion for downstream experiments.

Morphologically, the pEPSC‐derived endothelial cells exhibited cobblestone‐like monolayer structures, closely resembling the immortalized porcine endothelial cell line (PED) (Figure 4B, S9A). Transcriptomic profiling confirmed their endothelial identity. Unsupervised t‐SNE analysis revealed that pEPSC‐derived endothelial cells (WT‐endo and Q47‐endo) clustered tightly with the PED line, while clearly separated from undifferentiated pEPSCs and porcine embryonic fibroblasts (PEF) (Figure 4C). Heatmap analysis further revealed distinct molecular signatures among the three cell types: while pEPSCs retained pluripotency markers (*OCT4*, SOX2, NANOG, etc.) and PEFs expressed typical fibroblast genes (FN1, ACTA2, THY1, etc.), the endothelial clusters (WT‐endo, Q47‐endo, and PED) consistently upregulated vascular‐specific transcripts, including PECAM1 (CD31), CDH5 (VE‐cadherin), ENG (endoglin), and vWF (Figure 4D‐E). Gene Ontology (GO) enrichment analysis of highly variable genes in pEPSC‐derived endothelial cells identified significant enrichment for biological processes fundamental to vascular development (Figure 4F). Experimentally, flow cytometry analysis revealed over 90% of these pEPSC‐derived endothelial cells expressed CD31, akin to PEDs (Figure 4G, S9C). Collectively, these findings establish the successful generation of endothelial cells from porcine EPSCs for subsequent immunophenotyping.

### 3.5 PEPSC‐Derived Endothelial Cells Enable Rapid Immunophenotyping Assessment of Gene Edits

**FIGURE 5:**
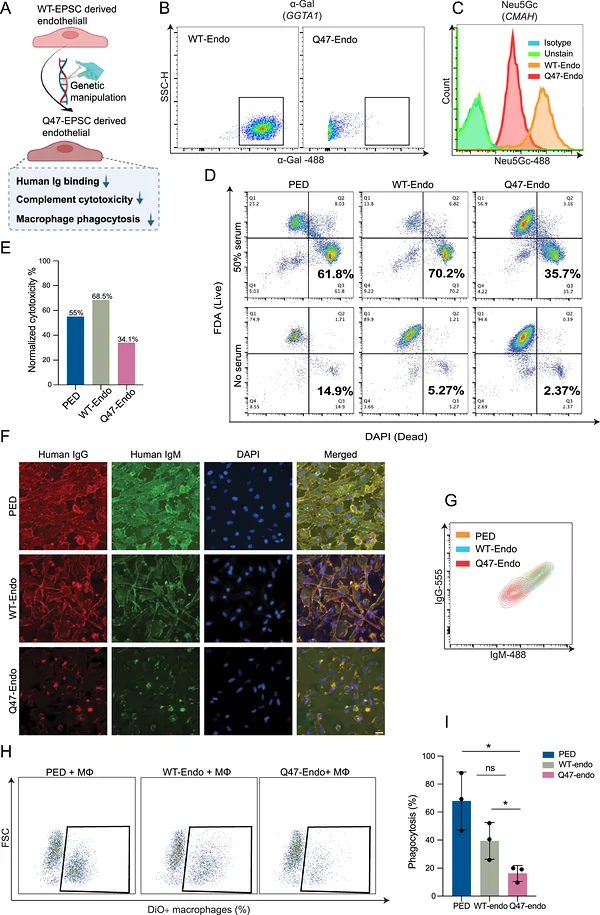
In vitro immunophenotyping of pEPSC‐derived endothelial cells. (A) Diagram showing pEPSC‐derived endothelial cells for immunophenotyping and assessment. (B) Flow cytometric analysis of α‐Gal (*GGTA1*) expression on pEPSC‐derived endothelial cells (WT‐Endo and Q47‐Endo). (C) Flow cytometric analysis of Neu5Gc (*CMAH*) expression on WT‐Endo and Q47‐Endo. (D) Flowcytometry assessment of complement‐mediated antibody‐dependent cytotoxicity in WT‐Endo and Q47‐Endo cells, under 0% or 50% pooled human serum. Live cells were stained with fluorescein diacetate (FDA, green), while dead cells were stained with DAPI (blue). PED were included as the positive control to evaluate the extent of cytotoxicity. (E) The normalized cytotoxicity result obtained from (D) was summarized in column chart. (F) Immunofluorescence detection of human IgG (red) and IgM (green) antibody binding to WT‐Endo and Q47‐Endo cells. PED were served as the positive control. Nuclei were counterstained with DAPI (blue). Scale bars: 25 µm. (G) Similar human antibody binding results were obtained by flow cytometry. (H) Flow cytometry assessment of human macrophage (Mφ) phagocytosis activity in Q47‐Endo, compared to WT‐Endo. PED were included as positive control. (I) The Mφ phagocytytosis activity across PED, WT‐Endo and Q47‐Endo was summarized in column chart. Error bars indicate ± SD (*N* = 3, independent experiments). ns, *P* ≥ 0.05. *P  <  0.05. Statistical analysis was performed using unpaired Student's *t*‐test.

Following successful derivation of endothelial cells from WT‐ and Q47‐pEPSCs, we evaluated xeno‐antigen expression, examined hyperacute rejection, and assessed macrophage phagocytosis (Figure 5A). While undifferentiated pEPSCs exhibited the Sd(a) epitope (Figure 3C, S8A), they lacked the glycan epitopes α‐Gal and Neu5Gc (Figure 3C, S8A). In contrast, abundant xeno‐antigen epitopes, particularly α‐Gal and Neu5Gc, were detected on the pEPSC‐derived endothelial cells in flow cytometry (WT‐endo in Figures 5B, 5C). We next evaluated the consequence of knocking out *GGTA1* (α‐Gal) and *CMAH* (Neu5Gc) in flow cytometry using the pEPSC‐derived endothelial cells. Compared to 96.6% positive for α‐Gal on WT‐endo (Figure 5B), α‐Gal was detected on only 2.55% of Q47‐endo. In line with *CMAH* inactivation, Q47‐endo also showed a substantial reduction of Neu5Gc (Figure 5C).

We next conducted a complement‐mediated cytotoxicity assay, a key mechanism of immune rejection in xenotransplantation [63]. The complement system, a cascade of proteins in the immune system, can be activated by antibodies binding to cell surfaces forming a membrane attack complex that creates pores in cell membrane, leading to cell lysis and death [64]. To simulate antibody‐dependent complement cytotoxicity, porcine endothelial cells were incubated with heat‐inactivated human serum which contains pre‐formed antibodies against glycan epitopes on cell surface. Complement components were then added to the incubated cells, followed by a live/death flow cytometry assay. Compared to WT‐endo (68.5%) and PED (55%), Q47‐endo exhibited a substantially lower cytotoxicity (34.1%) (Figures 5D, 5E). The gene edits in Q47‐pEPSCs therefore appear to confer an enhanced protection against complement‐mediated cytotoxicity in endothelial cells.

We subsequently used immunofluorescence and flow cytometry and investigated binding of human immunoglobulins (IgG and IgM) to pEPSC‐derived endothelial cells, which was detected using fluorochrome‐labelled secondary antibodies specific to human IgG and IgM. Consistent with the above complement assay, compared to WT‐endo, Q47‐endo showed significantly reduced binding of human immunoglobulins (Figures 5F, 5G), which further supports the protective effect of the gene edits in Q47‐pEPSCs in reducing antibody‐mediated immune rejection.

We next assessed the protective role of transgenic human CD47 against macrophage phagocytosis in an in vitro co‐culture assay. While human CD47 serves as a “don't eat me” signal by interacting with macrophage SIRPα to inhibit phagocytosis, due to interspecies incompatibility (73% amino acid identity), porcine CD47 cannot mediate downstream SIRPα phosphorylation in human macrophages, failing to transmit the inhibitory signal [65]. This deficiency underscores why unmodified porcine cells remain susceptible to phagocytosis in transplant recipients. To test the transgenic human CD47 function, endothelial cells derived from pEPSCs (Q47‐endo, WT‐endo) and PED were co‐cultured with human macrophages for phagocytosis (Figures 5H, 5I and S10). Co‐culture of porcine endothelial cells with human macrophages revealed that PED exhibited the highest phagocytosis rate (46.6%), followed by WT‐endo (40.5%). In contrast, Q47‐endo, expressing human CD47, showed significantly reduced phagocytosis (19%) (Figures 5H, 5I), confirming that human CD47 expression provides effective protection against macrophage phagocytosis.

## 4 Discussion

Xenotransplantation using genetically modified porcine organs has emerged as a promising solution to address the global organ shortage. Despite significant advances in this area, porcine fibroblast‐based systems face key limitations: (i) low efficiency for multiplex or complex editing [45, 48]; (ii) limited differentiation potential to produce highly immunogenic cells for rapid and quantitative immunophenotyping to evaluate and identify immunocompatibility genome edits.

Compared to gene editing in fibroblast requiring flow cytometry/magnetic bead sorting for single‐cell isolation and genotyping [66, 67], a process prone to contamination and demanding prolonged culture establishment, pEPSCs allow direct single cell clonal expansion, rapid genotyping and phenotyping (Figure S11) [51, 52]. Through a single‐round electroporation, we achieved simultaneous knockout in pEPSCs of three hyperacute rejection‐associated genes (GGTA1: 11% homozygous, CMAH: 33% homozygous, and B4GALNT2: 4% homozygous; Figure 1B), demonstrating efficient multiplex genome editing.

pEPSCs enable efficient precision genome editing. We established an RMCE [53] master pEPSC line by targeting *lox* docking sites to the *ROSA26* safe harbor locus. This approach allows for the easy and highly efficient introduction of multiple transgenes at a defined genomic locus, simplifying future breeding process. Additionally, RMCE can accommodate large DNA sequences for long transgenes, up to 300 kb [68], with minimal risk of genomic disruptions. By utilizing RMCE in porcine EPSCs, we achieved high efficiency (close to 100%) in integrating genes of interest, such as *EGFP* (data not shown) and human *CD47*, at the *ROSA26* locus. Although RMCE has been successfully performed in fibroblasts [11], the porcine fibroblasts carrying the correctly targeted RMCE cassette have to be used for generating cloned embryos from which second generation fibroblasts can be subsequently used for introducing human cDNAs. In contrast, pEPSCs carrying the RMCE cassette at a defined locus can be directly used for inserting human cDNAs directly.

pEPSCs are pluripotent stem cells and can generate highly immunogenic cells such as endothelial cells, which we used for immunophenotypic assessment of the edited cells. While traditional approaches require months of pig cloning using edited fibroblasts and the subsequent endothelial cell derivation [20, 21], we demonstrated that genome‐edited pEPSC‐derived endothelial cells in vitro recapitulated key immunological features related to xenotransplantation. These porcine endothelial cells exhibited significantly reduced complement‐mediated cytotoxicity (34.1% compared to 68.5% in wildtype cells), human immunoglobulin binding, and macrophage phagocytosis (19% vs. 40.5%). Importantly, besides endothelial cells, pEPSCs can also generate all other cell types, including cardiac, hepatic, pancreatic, and hematopoietic lineages, for immunophenotyping and for functional evaluation.

In conclusion, our data demonstrate that pEPSCs offer an efficient and versatile platform for multiplex genome editing and rapid in vitro immunophenotypic assessment, overcoming several key limitations of current fibroblast‐based approaches. This is evidenced by achieving quadruple gene knockouts and precise hCD47 knock‐in, and quick differentiation into endothelial cells for functional validation, which provides a more streamlined workflow for optimizing genetically engineered pig donors. Future studies will focus on generating live pigs from these gene‐edited pEPSCs via somatic cell nuclear transfer (SCNT) and exploring stem cells derived from miniature pig breeds to further improve organ size compatibility for xenotransplantation.

## 5 Materials and Methods

### 5.1 Porcine EPSCs

We used the porcine EPSC PK7 cell line, originally established by Xuefei and Monika from German Landrace pigs [51]. The PK7 porcine EPSCs were cultured on SNL (76/7) feeder cells following the published protocol [51, 52], with minor modifications. Briefly, the EPSC medium was consisted of an N2B27 basal medium supplemented with the following components: 0.2 µM CHIR99021 (Tocris, catalog no. 4423), 0.02 µM A419 (Tocris, 3914), 2.5 µM XAV939 (Sigma, X3004), 65.0 µg/ml Vitamin C (Sigma, A8960), 10.0 ng/ml LIF (Peprotech, 300‐05‐50UG), and 20.0 ng/ml Activin (Peprotech, AF‐120‐14E‐50UG). Passaging of the cells was performed every 3 to 5 days, with a typical passage ratio of 1:10 to 1:20.

The N2B27 basal medium was prepared as follows: 50% DMEM/F12, 50% Neural Basal, 0.5% N2, 1% B27, 1% ITS‐X, 1% Non‐Essential Amino Acids (Gibco, 11140050), 1% Penicillin‐Streptomycin‐Glutamine (Gibco, 10378016), 1 mM Sodium Pyruvate (Gibco, 11360070), 55 µM 2‐mercaptoethanol (Gibco, 21985023), 0.045% Bovine Albumin Fraction V (Gibco, 15260037), 0.2% Chemically Defined Lipid Concentrate (Gibco, 11905031), 3.3 µg/mL L‐Glutathione reduced (Sigma, G4251), 0.1% Trace Elements B (Corning, MT99175CI), 0.1% Trace Elements C (Corning, MT99176CI).

### 5.2 GRNAs and Genotyping Primers

CRISPR/Cas9‐mediated genetic knockout was performed in porcine EPSCs targeting (*GGTA1*, *CMAH*, *B4GALNT2*, and *GHR*). Two sets of sgRNA were designed to generate band shifting to perturb gene functions. The genotyping primers were designed to amplify the targeting region and detect band shifting via agarose gel electrophoresis between wild‐type and edited EPSCs. The genomic sequences were downloaded from the Ensembl database (www.ensembl.org), and a targeting region of interest within the genome would be uploaded to CRISPOR (http://crispor.tefor.net/) to select the sgRNA with high specificity and low off‐target score.

The knockin guide RNA and homologous arm (HA) sequence design for foreign gene insertion at the porcine ROSA26 locus was detailed by Gao. et al. [51]. The 5' and 3' junction PCR primers were used to detect the successful integration.

The guide RNAs were ordered and synthesized via Synthego (https://www.synthego.com/). The genotyping primers were ordered from IDT. Refer to Table S1 for gRNA sequences and Table S2 for genotyping primer sequences.

### 5.3 Electroporation

The porcine EPSCs was refreshed with culture medium supplemented with 10% FBS around two or three hours before electroporation. Approximately 1 to 2 × 10^5^ porcine EPSCs were used in a 10 µL Neon transfection system (Invitrogen, MPK1025). Porcine EPSCs were digested by TrypLE (Gibco, 12604021) at 37°C for 5 min, followed by gentle pipetting to dissociate into single cells. Porcine EPSCs/feeder mixture were then flowed through a 40 µm cell strainer to remove most feeders and obtain a single EPSC suspension. EPSC cell number was counted and adjusted at a density of 2 to 4 × 10^7^ in Resuspension buffer R (Invitrogen, MPK1025). Total 5 µL solution containing 1 to 2 × 10^5^ EPSCs in Resuspension buffer R was transferred to a 1.5 mL tube, which already contained another 7 µL DNA in Resuspension Buffer R, making a total volume of 12 µL for one electroporation. For knockout experiments, the DNA mixture contained 400 ng Cas9 protein (Thermo, A36498), two sets of 1.2 pmol sgRNA (sgRNA synthesized from Synthego). For knock‐in purposes, we used 200 ng Cas9 protein, 1.2 pmol sgRNA, and 1.5–3 µg donor plasmid. The total volume of Cas9 protein, sgRNA, and donor plasmid was less than 1 µL. For Cre‐mediated cassette exchange, we used 1 µg Cre‐mCherry plasmid, and 500 ng ‐ 1 µg donor DNA. We set the electroporation parameters at 1200 V, 20 ms, 2 pulses for porcine EPSCs. Then the electroporated EPSCs were seeded in two feeder wells prewarmed with 10% FBS in EPSC medium plus 10 µM Y27632 (MCE, HY‐10071). Then we changed to normal EPSC culture medium the next day. The single electroporated porcine EPSC‐derived colonies would be picked around day 10 to day 12.

### 5.4 Bulk Genotyping After Electroporation

One week after electroporation, when multiple colonies have emerged, we collected one well of cells for genomic DNA extraction. Wild‐type genomic DNA was included as a control. We amplified the genotyping PCR with bulk and control samples and ran gel electrophoresis to analyze the band sizes between the bulk sample and the wildtype control to assess the general gene‐editing efficiency. For knockout experiments using two sgRNAs, two bands were expected, with the upper band representing the wildtype band and the lower band representing the knockout band. The brightness of the knockout band can indicate the general knockout efficiency. For knockin experiments, we used bulk genomic DNA to test various junction PCR conditions. Wild‐type genomic DNA was included as a control. The right band size indicated the successful integration of the foreign cassette.

### 5.5 Colony Picking and Sanger Sequencing

Single EPSC colonies were manually picked around day 10–12 post‐electroporation, when they were visible to the naked eye and discrete under a dissection microscope. We decided the colony number to be picked based on the bulk genotyping results.

Colony picking was performed in a biosafety flow hood on a 37°C heating pad using a dissection microscope. Prior to picking, the culture medium was replaced with DPBS to facilitate colony detachment. Individual colonies were gently scratched and lifted with a sterile 10 µL tip under microscopic, ensuring only discrete, non‐contiguous colonies were selected to avoid cross‐contamination from neighboring cells. Each picked colony was transferred into a 20 µL drop of TrypLE supplemented with 10 µM Y27632 in a 96‐well plate and incubated for 3 min at room temperature. The colony was then dissociated into small clumps by gentle pipetting. Half of the dissociated colony was lysed directly (using NP‐40 lysis buffer) as template for genotyping PCR, while the other half was replated onto fresh feeder cells for expansion.

Genomic editing outcomes in the lysed half were initially screened by PCR, and positive clones were further validated by Sanger sequencing of the PCR products to confirm homozygous knockouts and absence of unintended edits.

### 5.6 Fast Genotyping Template Preparation From Single Colonies

NP‐40 lysis buffer was prepared as follows: To make 10 mL, mix 400 µL of 10% NP‐40 (Thermo Fisher, 28324), 500 µL Proteinase K (Takara, 9034), and 9.1 mL ultra‐pure water (Thermo Fisher, 10977023) in a 15 mL falcon tube by gentle inversion. The buffer was aliquoted (1 mL per vial) and stored at ‐20°C for up to 6 months.

For template preparation, half of each manually picked colony was lysed by adding 20 µL of NP‐40 lysis buffer. Samples were briefly spun down and subjected to the following thermocycler program: 56°C for 1 h, 95°C for 10 min, followed by a 4°C hold. The resulting lysate was used directly as PCR template or stored at 4°C for up to 2 weeks or at ‐20°C for up to 6 months.

### 5.7 Polymerase Chain Reaction (PCR)

Genomic DNA or NP‐40 lysis product was used as the template for PCR amplification. A touch‐down PCR program was used for most amplifications unless otherwise specified, with annealing temperature starting at 68°C and decreasing by 0.6°C per cycle over 35 cycles (other steps followed the standard protocol for each polymerase).

High‐fidelity amplification for Sanger sequencing validation was performed using PrimeSTAR Max DNA Polymerase (Takara Bio, R045A). Rapid genotyping assays (initial screening of colony lysates as mentioned above) were performed using Rapid Taq Master Mix (Vanzyme, P222). PCR reactions were prepared on ice and run on thermal cyclers.

### 5.8 RMCE Vector for Targeting to the *ROSA26* Locus

The foreign cassette was flanked by *lox66* (5') and *lox2272* (3') containing the PuroΔTK driven by CAG promoter. The pig *ROSA26* 5' and 3' homologous arms were previously cloned into pRosa26‐EF1a‐H2b‐mCherry vector. Therefore, by replacing the EF1a‐H2b‐mCherry fragment with CAG‐ PuroΔTK flanked by *lox66* and *lox2272* cassette, the RMCE donor vector could be used for targeting (Table S7). The pRosa26‐EF1a‐H2b‐mCherry vector was kindly provided by Dr. Xuefei Gao [51].

### 5.9 Cre‐Mediated Cassette Exchange for Inserting Human *CD47* cDNA Into the Pig *ROSA26* Locus

The human *CD47* expression cassette was PCR amplified from the 4Tg plasmid (kindly provided by Dr. Björn Petersen). The cassette consists of the CAG promoter, a chimeric intron, the full‐length coding sequence of human CD47 isoform 1, and a Rabbit β‐globin polyA signal. To allow for the Cre‐mediated cassette exchange, 34 bp overhangs with *lox* sequences were designed to make long PCR primers. The forward primers had the *lox71* sequence: ATAACTTCGTATAATGTATGCTATACGAACGGTACCAGATATACGCGTTGATCTCC (5' to 3'). The reverse primers contained the *lox2272* sequence: ATAACTTCGTATAGGATACTTTATACGAAGTTATGCTAGCTGAGGATCGATACGAC (5' to 3'). The PCR‐amplified human *CD47* exchange cassette was further analyzed by agarose gel and confirmed by Sanger sequencing. The amplified PCR product was further purified by a spin column for targeting (TIANGEN, 4992196). The detailed sequence map of the human *CD47* donor cassette is provided in Figure S4A and Table S7.

### 5.10 Drug Selection for Rmce

For positive selection of RMCE integration, we used 2 µg/ml puromycin (InvivoGen, ant‐pr‐1) to enrich the correctly targeted clones. For negative selection during human CD47 exchange at the *ROSA26* locus, we used 2 µM Ganciclovir (MCE, HY‐13637). The drug exposure was maintained for 2 days after electroporation and then we switched to normal EPSC culture medium.

### 5.11 Glycan Epitope Staining for Immunofluorescence and Flow Cytometry

For immunofluorescence (IF): Cells were washed with HBSS (Gibco, 14025076), fixed in 4% paraformaldehyde (PFA), blocked in 1% BSA in HBSS except for Neu5Gc (which used the kit‐provided Neu5Gc Assay Blocking Solution), and stained with lectins or antibodies. Cells were counterstained with DAPI for IF and examined under a Nikon Eclipse microscope.

For flow cytometry: Live cells were harvested and stained with lectins or antibodies in HBSS‐based FACS buffer, except for Neu5Gc (which used the kit‐provided Neu5Gc Assay Blocking Solution for pre‐incubation, followed by staining per the manufacturer's protocol). Flow cytometry was performed on an ACEA NovoCyte Quanteon, with data analyzed using FlowJo software.

α‐Gal was stained by FITC‐conjugated Isolectin B4 (Sigma, L2895) for flow cytometry, or DyLight 594‐conjugated Isolectin B4 (Vector Laboratories, DL‐1207‐.5) for immunofluorescence, both at 1:100 dilution. Sda was stained using FITC‐conjugated Dolichos biflorus agglutinin (DBA, Invitrogen, L32474) at 1:100 dilution for IF and flowcytometry. The expression level of Neu5Gc was assessed by the Anti‐Neu5Gc Antibody Kit (BioLegend, #146901), following the manufacturer's protocol for flow cytometry and immunofluorescence.

### 5.12 Human Antibody Binding Assay

Pooled normal human male AB serum (Innovative Research, ISER50ML) was heat‐inactivated at 56°C in a water bath for 30 min and centrifuged at 1000 rpm for 5 min to remove solid components. Then, the serum was diluted in FACS buffer (0.2% BSA, 5 mM EDTA in DPBS) and incubated with porcine EPSC‐derived endothelial cells for 30 min at room temperature. After rinsing with cold HBSS three times, the cells were incubated with diluted anti‐human IgG‐555 (Thermo Fisher, A‐21433) and anti‐human IgM‐488 (Thermo Fisher, A‐21215) at room temperature for 30 min, followed by DAPI for nuclear staining. For flow cytometry, cells were analyzed with ACEA NovoCyte Quanteon and FlowJo software; alternatively, for imaging, cells were fixed with 4% PFA before fluorescent image capture (Nikon Eclipse). This protocol was adapted from Yue. et al. [21].

### 5.13 Complement‐Mediated Cytotoxicity Test

The targeted cells were incubated the heat‐inactivated human serum (diluted 50%) with at 4°C for 30 min. A control group without human serum was included to establish background cell viability. After rinsing with cold HBSS three times, the cells were incubated with the diluted baby rabbit complement (Bio‐Rad, C12CA.1) in FACS buffer at a 1:5 ratio. The cell/complement mixture was incubated at 37°C for 30 min. After washing step, the cells were resuspended in 100 µL of cold Live/Dead staining solution containing 1 µg/mL FDA + 0.1 µg/mL DAPI.

The samples were run in the ACEA NovoCyte Quanteon flow machine. The quadruple gating was employed based on FDA and DAPI staining to distinguish between live and dead cells. Dead cells were defined as FDA‐negative and DAPI‐positive. The cytotoxicity score was calculated according to the formula below: Cytotoxicity (%) = (% Dead cells with serum—% Dead cells no serum) / (100 ‐ % Dead cells no serum).

### 5.14 Macrophage Phagocytosis Assay

THP1 differentiation into M1 macrophages was achieved by a 24 h treatment with 150 nM PMA, followed by a 48 h exposure to 10 ng/ml LPS and 25 ng/ml IFN‐γ. Subsequently, target cells (porcine EPSC‐derived endothelial cells; PED) were labeled with DiOC (Thermo Fisher, L7010) by adding 2 µL of DiOC per 1 mL of culture medium and incubating them at 37°C overnight. After DPBS washing, the labeled cells were collected, counted, and resuspended at a concentration of 10^6^ cells per 1000 µL of M1 medium. For coculture experiments, DiOC‐labeled target cells were cocultured with an equal number of macrophages (ET ratio 1:1) at 37°C for 4 h. Two control groups, including target‐only and effector‐only, received the same treatment. The macrophages were then labeled with CD11b‐APC‐Cy7 antibody (BD, 560914) in the dark for 30 min on ice, followed by live cell staining with DAPI. Flow cytometry was performed using the ACEA NovoCyte Quanteon, and FlowJo software was used for subsequent analysis.

### 5.15 Human Macrophages

Macrophages used in the phagocytosis assay were derived from THP1 cells, generously provided by Dr. Rio Sugimura. THP1 cell maintenance followed ATCC recommendations (https://www.atcc.org/products/tib‐202). These cells were cultured in RPMI‐based medium (Gibco, Cat: 22400–71) containing 10% heat‐inactivated FBS (Gibco, cat. no. 10270106), 200 g/L glucose (Gibco, Cat: A24940‐01), 1 mM sodium pyruvate (Gibco, Cat: 11360070), and 55 µM 2‐mercaptoethanol (Gibco, Cat: 21985023). The seeding density was from 2 to 5 × 10^5^/mL, with medium replacement every 2 to 3 days. Passage was typically performed when cells reached 1 to 2 × 10^6^/mL, with a split ratio of 1:4 to 1:6. Confluency was usually achieved within 3 to 5 days post‐passage. Cells were maintained at 5% CO_2_ and 37°C.

The differentiation from THP1 to macrophage was performed when THP1 reached the logarithmic growth phase. Add 150 nM PMA to THP1 culture medium for 24 h. This transition from suspension to an attached state signifies the macrophage status. These attached macrophage cells can be polarized into the pro‐inflammatory M1 subtype by introducing 20 ng/mL IFN‐γ and 10 pg/mL LPS into the THP1 medium (without PMA) for 2 days. Once polarized in M1 medium, these cells are suitable for phagocytosis assays.

### 5.16 Porcine Endothelial Cell (PED) Line

The immortalized porcine endothelial cell line PED‐SV15 (hereafter referred to as PED) was generously provided by Dr. Björn Petersen at the FLI institute in Germany. The PED cells were cultured in endothelial cell expansion medium (STEMCELL Technologies, 08005) on Matrigel‐coated dishes for immune phenotyping experiments. Alternatively, the cells were cultured in DMEM (Gibco, cat. no. 11995073) supplemented with 10% FBS (Gibco, cat. no. 10270106), 1% Penicillin‐Streptomycin (Gibco, 15140122), 1% Non‐essential amino acids (Gibco, 11140050), 90 µg/ml ECGF (ReliaTech, 300‐092) for daily maintenance. The medium was refreshed every other day. When the cells reached 90% to 100% confluency, they were passaged at a ratio of 1:2 to 1:5. For seeding, approximately 3 × 10^5^ cells were used per well of a 6‐well plate.

### 5.17 Derivation of Endothelial Cells From pEPSCs

To initiate the differentiation of porcine endothelial cells, 5000 pEPSCs without feeder cells were harvested and placed onto 1% Matrigel‐coated 12‐well plates (Matrigel, Corning, 356231). Subsequently, the pEPSCs were primed in Essential 8 medium (Gibco, A1517001) for 48 h. Following the priming phase, the cells transitioned to the “post‐implantation”‐like stage, referred to as Day 0.

The primed pEPSCs were directed towards mesoderm differentiation by exposing them to mesoderm induction medium (STEMCELL Technologies, 05220) for 48 h without disturbance. Subsequently, on Day 2, the cells were further guided towards the endothelial lineage through the utilization of endothelial cell induction medium (STEMCELL Technologies, 08005), which was maintained for an additional 4 days, with medium changes every other day.

By Day 6, these cells had effectively committed to the endothelial lineage, making them suitable for either subculturing or more extensive analysis. During this period, their CD31 positivity was evaluated via flow cytometry. These endothelial cells, coexisting with other miscellaneous cell types within the culture, were identified as passage 0 (P0) and could be further expanded utilizing endothelial expansion medium (STEMCELL Technologies, 08007). The endothelial cells generated through this protocol were readily applicable for immunophenotyping assays. Furthermore, these cells could undergo multiple passages and freeze‐thaw cycles, maintaining their integrity up to passage 8 without noticeable differences. When the cells reached full confluence, they could be passaged onto Matrigel‐coated plates, with a passage ratio of 1:3. However, an extended passage number might lead to a decrease in their proliferative capacity.

### 5.18 PEPSC Feeder Removal for Endothelial Differentiation

The removal of feeder cells followed established pEPSC passage protocols, with slight modifications as previously detailed by Gao. et al. [51]. This procedure is crucial for isolating pure porcine EPSCs and relies on the differential detachment characteristics observed when using detaching agents such as EDTA or ReLeSR (STEMCELL Technologies, 100–0483).

Feeder‐free pEPSCs were collected when they reach a confluence of 70%–90%. Initially, the cells were rinsed with DPBS, followed by the addition of 0.5 mM EDTA and incubation at 37°C for 7 to 10 min. Alternatively, ReLeSR could be added, followed by a 5 to 7 min incubation. Subsequently, the detachment of EPSC colonies from the culture plate was confirmed under a microscope. The gentle swirling and tapping of the plate's bottom facilitated the detachment process.

A P1000 pipette was used to carefully dispense an equivalent volume of M10 medium drop by drop, to further enhance detachment while simultaneously neutralizing the dissociation process. Some residual feeder cells may be present in the supernatant; however, this presence did not negatively impact the overall differentiation process

The supernatant containing EPSC colonies was collected into a 15 mL tube and subjected to centrifugation at 200 g for 2 min at room temperature. Following centrifugation, the resulting pellet should be resuspended in culture medium, preparing it for subsequent experiments.

### 5.19 Reverse Transcription Quantitative—PCR (RT‐qPCR)

RNA was isolated using the Trizol method (Invitrogen, 15596026). After quantification, 2 µg of RNA was reverse transcribed to cDNA in a 20 µL reaction using FastKing gDNA Dispelling RT SuperMix (TIANGEN, KR118‐02) with gDNA eraser activity. The diluted cDNA was used as template and amplified using SYBR Green (Applied Biosystems, A25743) in QuantStudio 7 System (Applied Biosystems). The gene expression was determined relative to *GAPDH*. Data were analyzed using the ∆Ct or ∆∆Ct method, and experiments were performed in triplicate.

### 5.20 Bulk RNA Sequencing and Analysis

Total RNA was extracted using the Trizol method, with 0.5 µg of RNA serving as input for library construction. Library quality was assessed using Qubit for quantification and a bioanalyzer for size distribution detection. Then libraries were pooled and sequenced on Illumina platforms based on effective library concentration and required data output.

Post‐sequencing, the RNA‐seq reads were processed with fastp software for quality trimming and then aligned to the Sus scrofa reference genome using Hisat2. Gene expression levels were quantified using FeatureCounts, with results presented in FPKM (Fragments Per Kilobase of transcript per Million mapped reads) to account for both sequencing depth and gene length. For the identification of differentially expressed genes, the DESeq2 R package was employed.

For visualization, the t‐SNE plot was generated using ggplot2 and the heatmap was plotted using the pheatmap package, both based on variance‐stabilizing transformed counts from DESeq2.

### 5.21 Karyotyping

The porcine EPSCs at 70%–80% confluency was refreshed with culture medium one hour before experiment. Then 1 µg/mL colcemid (Gibco, 15212012) was added to the culture medium at 37°C for 3 h. The cells were digested with TrypLE and filtered through a 40 µM cell strainer to remove most feeders and obtain single cells. Then the cells were resuspended with hypotonic solution (0.56% KCl in ddH_2_O) and incubated them at 37°C water bath for 10 min with intermittent shaking. After hypotonic treatment, 2 mL of pre‐cooled fixation buffer (methanol: acetic acid = 3:1) was directly added without pipetting to pre‐fix the cells. The cells were further centrifuged and resuspended in 6 mL fixation buffer with gentle flicking. The fixation was repeated for 3 times. For the last fixation, 100 µL of fixation solution was left to resuspend the cells. The cells in suspension were dropped onto the pre‐cooled slide for chromosomes spreading. After air‐drying overnight in room temperature, the slides were sealed with DAPI (BD, 564907). Finally, the slides were examined under the microscope (Zeiss ELYRA) and approximately 30 spreads were counted for further analysis.

### 5.22 Teratoma Formation

Around 2 wells of a 6‐well plate containing porcine EPSCs at 70% confluency were required. The EPSCs were selectively collected without feeders by filter through a 40 µM cell strainer. The collected EPSCs were resuspended in 100 µL of medium containing EPSC basal supplemented with 10 µM Y27632 and 30% Matrigel (Corning, 356231). The cell suspension was kept on ice until injection. The resuspended EPSCs were subcutaneously injected at both sides of the dorsal flank region of NSG mice (4 to 12 weeks old, female). Teratoma samples were collected when their size over 1.5 cm^3^, which typically took around 8–12 weeks. Gross images of the teratoma samples were taken, and then the samples were fixed in 4% PFA overnight at 4°C. Subsequently, they were transitioned to 70% ethanol for further processing involving paraffin embedding, sectioning, and H&E staining. The histological sample processing was conducted by The Department of Pathology at Queen Mary Hospital. All procedures involving animals in this study were approved by the Committee on the Use of Live Animals in Teaching and Research (CULATR) of The University of Hong Kong (Approval No. 4731‐18).

### 5.23 Embryoid Body (EB) Formation

Porcine EPSCs were harvested at approximately 70% confluency. The collected cells were then primed in E8 medium (Gibco, A1517001) or M10 medium for 2 days on Matrigel‐coated plates, with a seeding ratio of 1:1. During the first day of priming, 10 µM Y27632 (MCE, HY‐10071) was added to the medium. Following priming, the EPSCs were dissociated and resuspended in EB medium with 10 µM Y27632. The composition of the EB medium included 20% KnockOut Serum Replacement (KSR; Gibco, 10828028), 1% Non‐Essential Amino Acids (Gibco, 11140050), 1% Penicillin‐Streptomycin‐Glutamine (Gibco, 10378016), 55 µM 2‐mercaptoethanol (Gibco, 21985023), and 65.0 µg/ml Vitamin C (Sigma, A8960) in DMEM/F12 (Gibco, 21331020). The cells were transferred to a low attachment well (Corning, 3741) for EB formation for 2 to 4 days. The resulting EBs were collected by centrifugation at 100 g for 1 min. Subsequently, the EBs were seeded onto Matrigel‐coated plates for further culture and differentiation in EB medium, with 10 µM Y27632 added during the first 24 h of differentiation. EBs were collected for experiments after 1 to 2 weeks post‐attachment.

### 5.24 Cell Cycle Analysis

Cells were cocultured with 10 µM EdU for 1 h, fixed, permeabilized, and stained with 6‐FAM azide (pseudo‐colored green) using the BD Pharmingen 488 EdU Click Proliferation Kit (Cat. No. 565455) according to the manufacturer's protocol. Nuclei were counterstained with DAPI (blue). EdU‐positive cells (S‐phase) exhibited green fluorescence, while EdU‐negative cells (non‐S‐phase) showed only DAPI staining. DAPI intensity was used to distinguish G0/G1 (2N DNA content) from S/G2/M phases (higher DNA content). Samples were acquired on ACEA NovoCyte Quanteon flow cytometer. Data were analyzed using FlowJo.

### 5.25 Ki‐67 Staining for Flowcytometry

Cells in exponential growth were refreshed with complete medium 3–4 h before harvest. After dissociation, cells were fixed in 0.5% PFA at room temperature for 15 min, then resuspended in 1× BD Perm/Wash Buffer (BD, 554723). Intracellular staining was performed with APC‐conjugated anti‐Ki‐67 antibody (BioLegend, 350513) for 30–60 min. After wash steps, cells were stained with DAPI before flowcytometry (ACEA NovoCyte Quanteon). Data were analyzed using FlowJo.

### 5.26 Off‐Target Analysis

Potential off‐target sites for all sgRNAs were predicted using CRISPRseek (v1.51.0) [69] against the pig reference genome (Sscrofa11.1), allowing up to 3 mismatches. WGS was performed on WT‐ and Q47‐pEPSCs at approximately 40× coverage. INDELs were called using GATK v4.2.6.1 [70] with standard filtering parameters. Structural variants (SVs) were called using Manta v1.6.0 [71] with default parameters. A variant was considered an off‐target candidate if it was located within 10 bp of a predicted site, present in Q47‐pEPSCs but absent in WT‐pEPSCs, and manually confirmed using IGV [72].

### 5.27 Statistical Analysis

All statistical analyses were conducted using GraphPad Prism software. Normality of the data was tested using the Shapiro–Wilk test. When the data did not pass the normality assumption, the non‐parametric Mann–Whitney test was performed. The threshold for statistical significance was set at *p* < 0.05.

## Author Contributions

P.L envisioned and conceptualized the project, P.L and B.P supervised the project, Y.Y.X, Y.X, X.W performed the experiments, Y.X. and Y.Y.X. conducted data processing and analysis, Y.Y.X wrote the original manuscript, Y.Y.X, Y.X, B.P and P.L review and edited the manuscript.

## Funding

This project was supported by the National Key Research and Development Program of China (no. 2022YFA1105401); the InnoHK initiative of the Innovation and Technology Commission of the Hong Kong Special Administrative Region Government; HKSAR, Hong Kong Research Council (Germany/Hong Kong travel grant G‐HKU704/21); RGC General Research Fund (GRF) (17127219), RGC Collaborative Research Equipment Grant (C7063‐23E).

## Conflicts of Interest

The authors declare that a patent application related to the findings of this study has been filed on behalf of InnoHK Centre for Translational Stem Cell Biology. Apart from this, the authors have no other competing interests to disclose.

## Supporting information




**Supporting Information**: xen70161‐sup‐0001‐SuppMat.docx

**Supporting Information**: xen70161‐sup‐0002‐SuppMat.pdf
