# Supplementary material for: Porcine Expanded Potential Stem Cells as a Versatile Platform for Multiplex Genome Editing and Immunophenotyping in Xenotransplantation
Source: Xenotransplantation. 2026 Aug 21;33(4):e70161. doi: 10.1111/xen.70161 (PMC13494693; doi:10.1111/xen.70161)
Supplement: Supplementary file 1 [file XEN-33-e70161-s001.pdf]

Figure S1

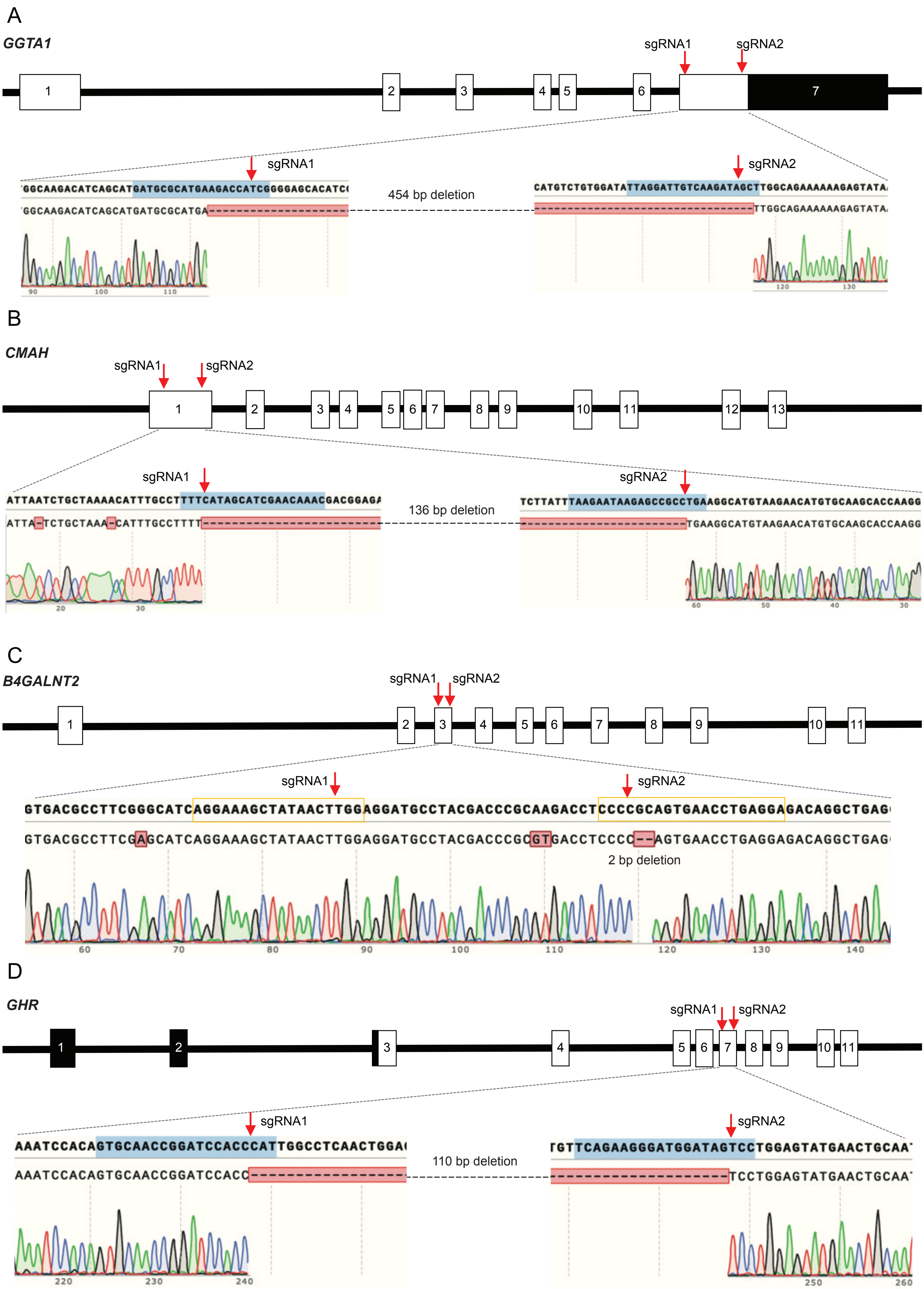
A
GGTA1
sgRNA1
sgRNA2
454 bp deletion
B
CMAH
sgRNA1
sgRNA2
136 bp deletion
C
B4GALNT2
sgRNA1
sgRNA2
2 bp deletion
D
GHR
sgRNA1
sgRNA2
110 bp deletion

Figure S2

A

GGTA1

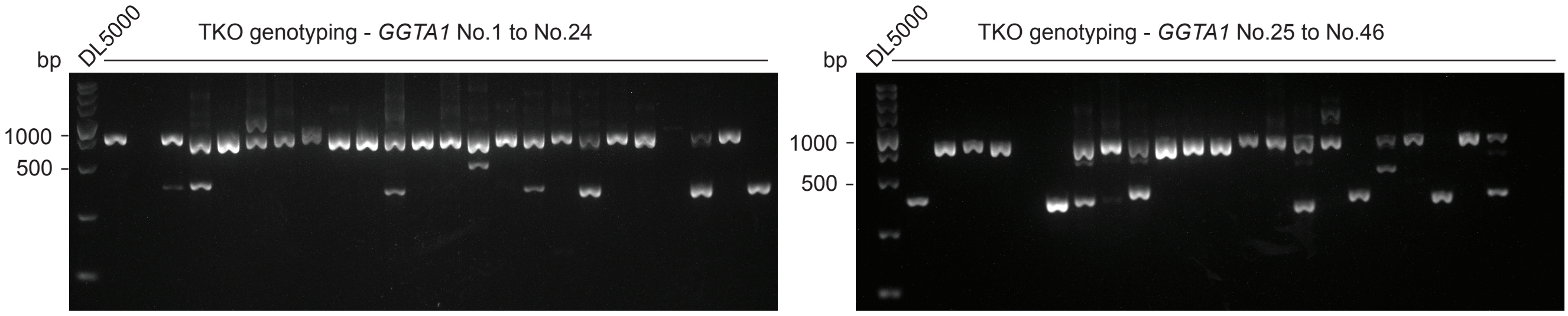

B

CMAH

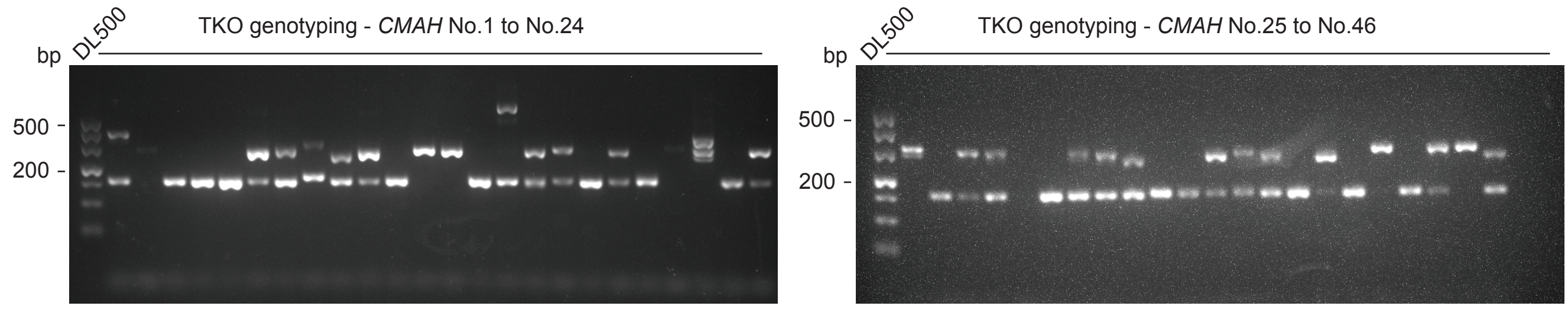

C

B4GALNT2

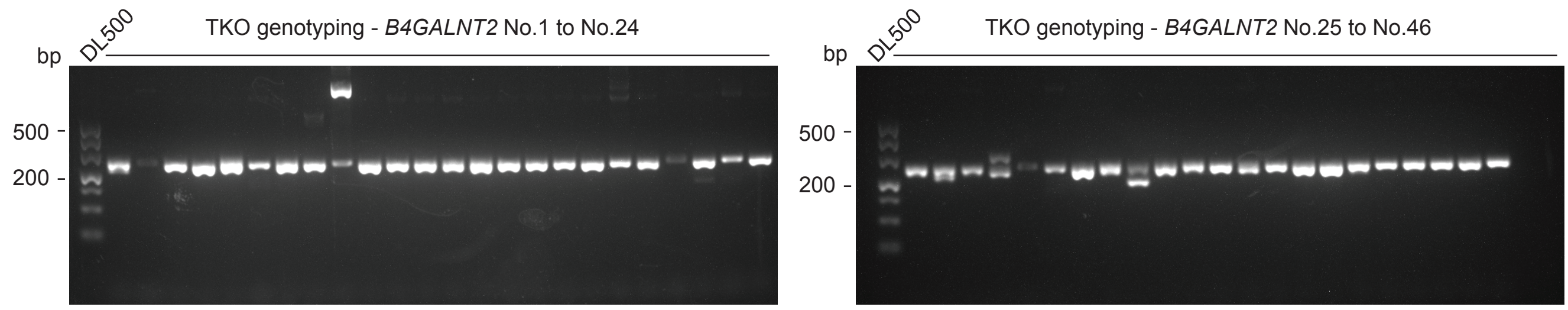

D

GHR

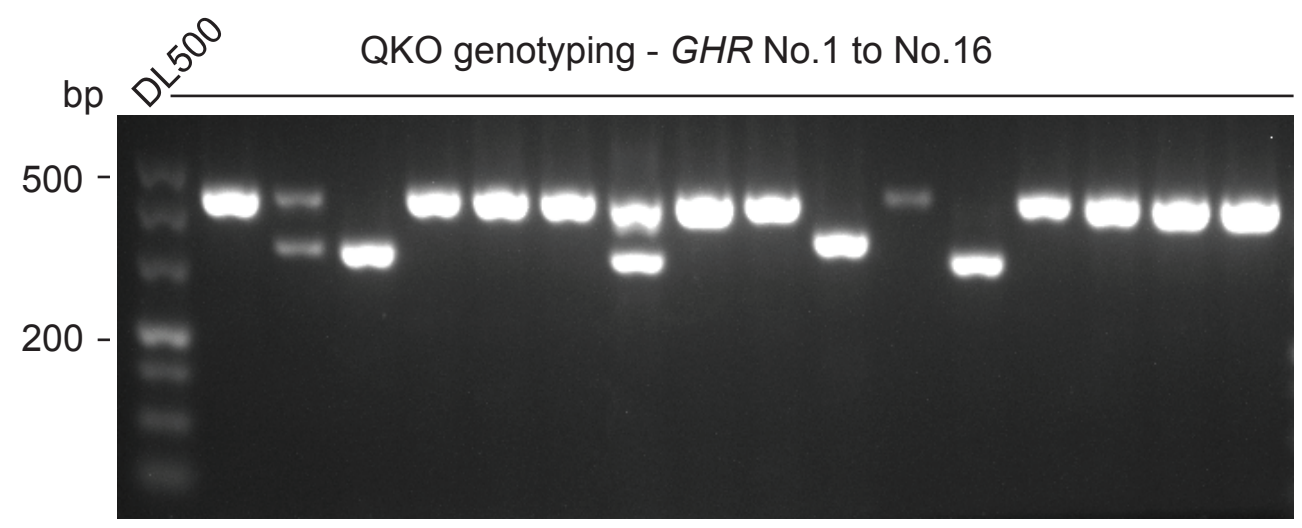

Figure S3

A

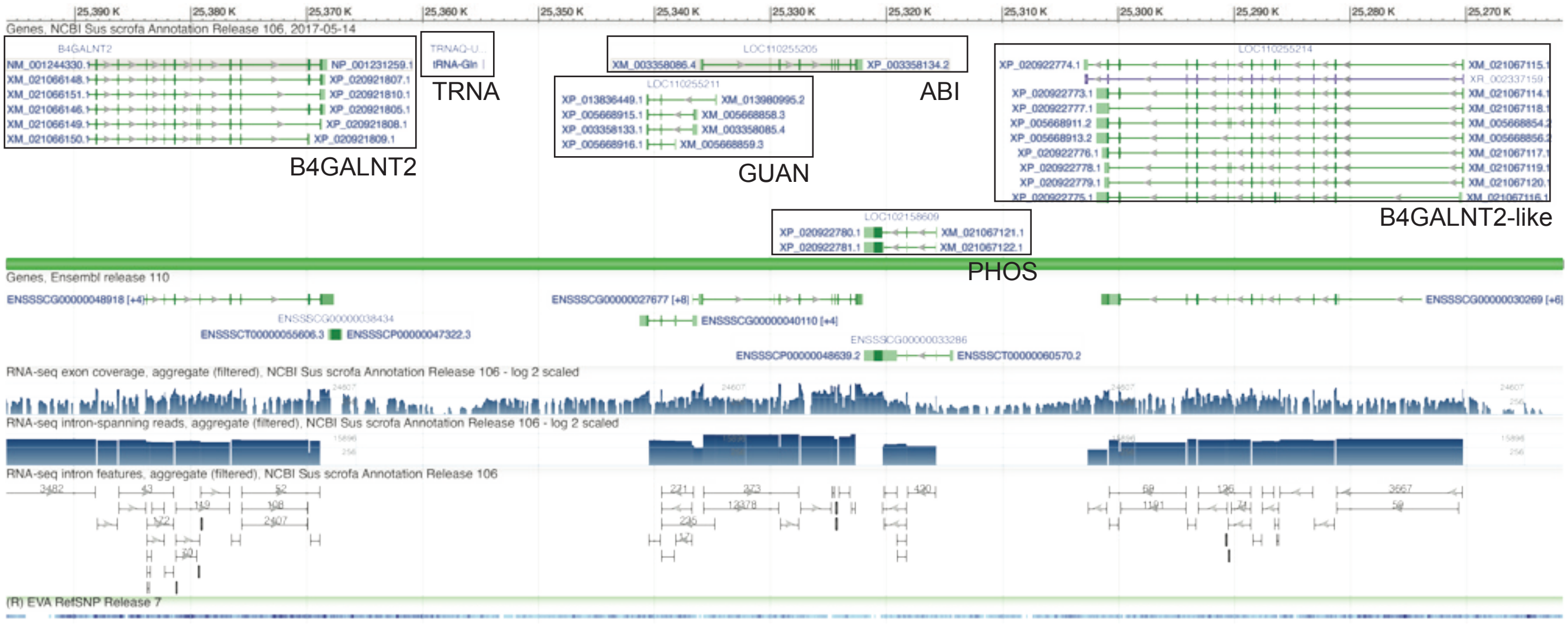

B

Gene symbol	Gene description	Abbreviation
LOC110255205	ABI gene family member 3-like	ABI
LOC102158609	phosphoethanolamine guanine nucleotide-binding	PHOS
LOC110255211	protein G(I)/G(S)/G(O) subunit gamma-T2	GUAN
TRNAQ-UUG	tRNA	TRNA

C

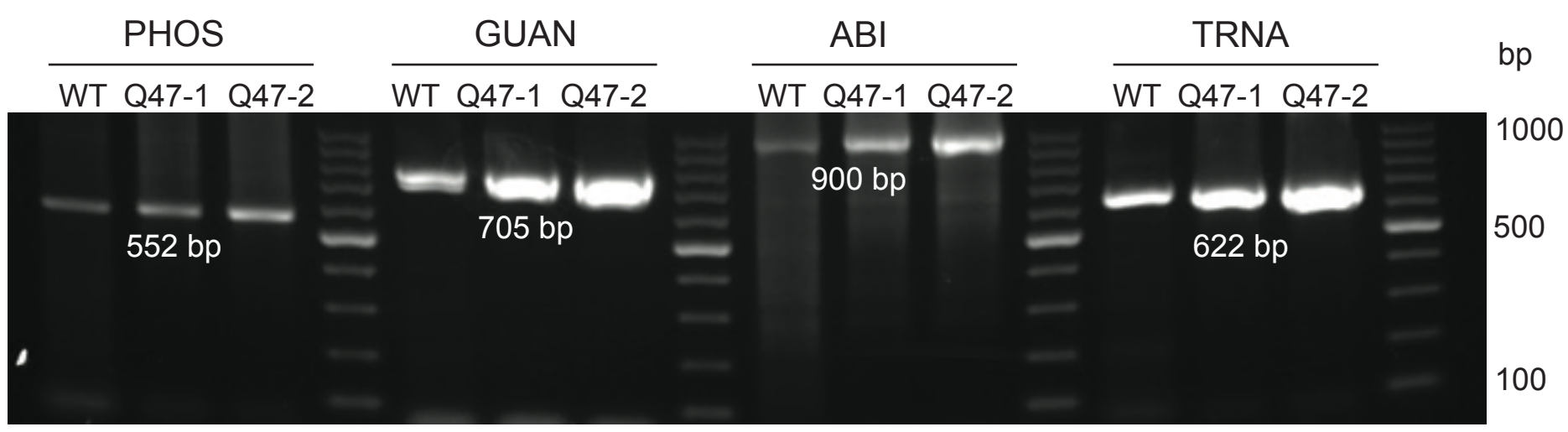

Figure S4

A

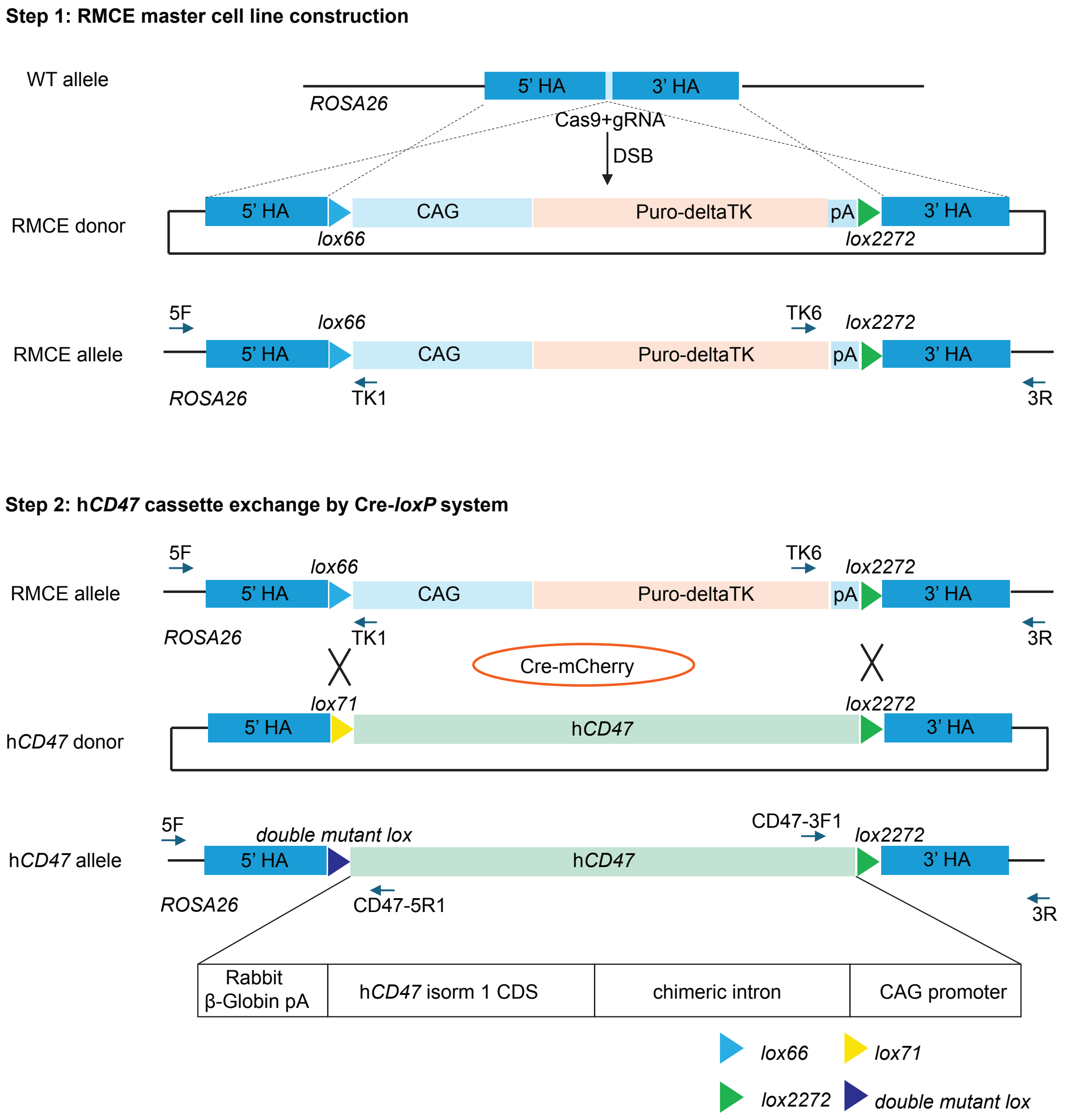

B

Exptected RMCE genotyping results

		Control	Targeted
5' Junction PCR	5F + TK1	--	1129bp
3' Junction PCR	TK6 + 3R	--	1485bp

Exptected h*CD47* genotyping results

		Control	Targeted
5' Junction PCR	5F + 5R1	--	814bp
3' Junction PCR	3F1+ 3R	--	848bp

Figure S5

A

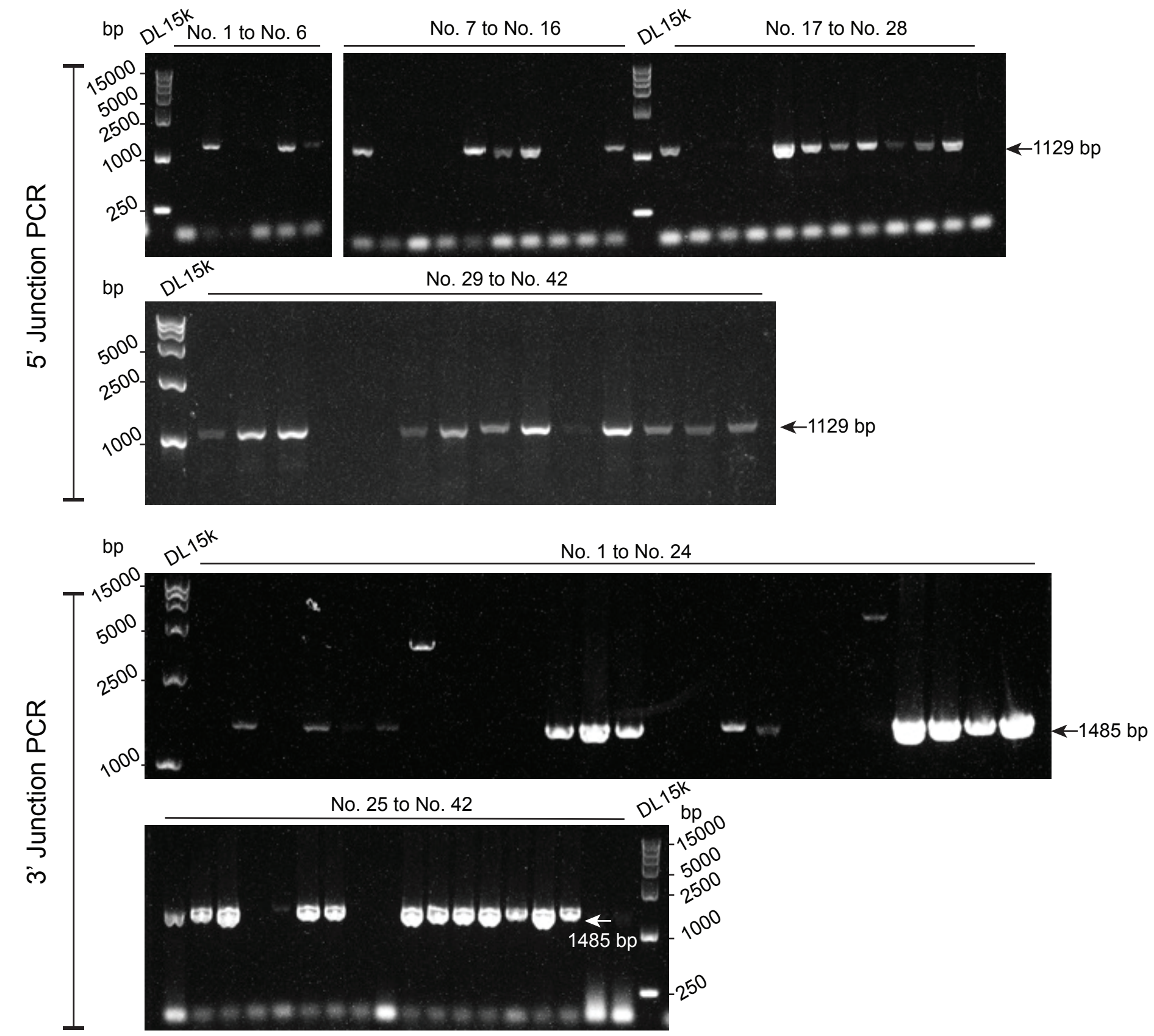
bp
DL15k
No. 1 to No. 6
No. 7 to No. 16
No. 17 to No. 28
15000
5000
2500
1000
250
←1129 bp
5' Junction PCR
No. 29 to No. 42
←1129 bp
No. 1 to No. 24
←1485 bp
3' Junction PCR
No. 25 to No. 42
1485 bp

B

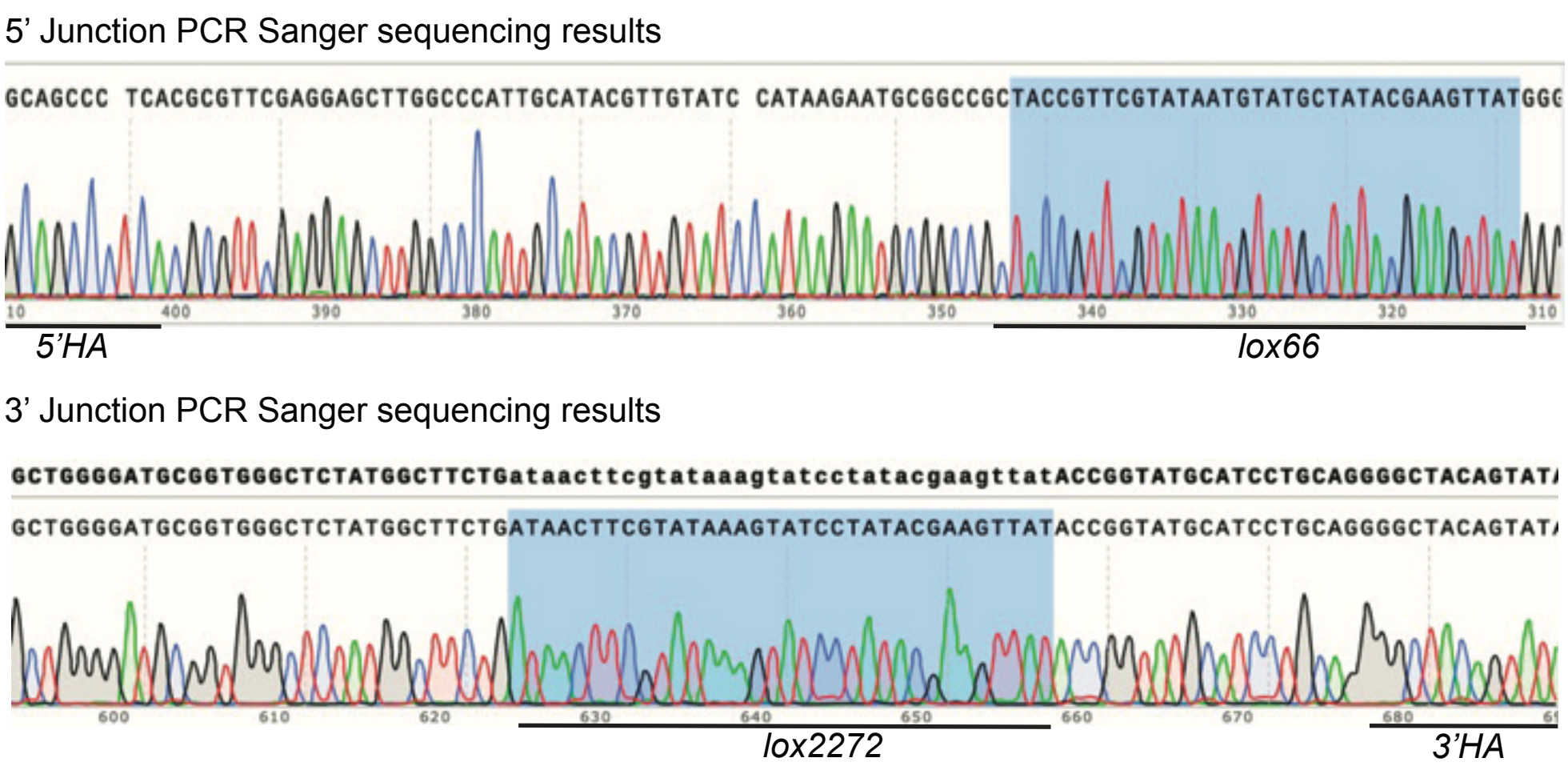
5' Junction PCR Sanger sequencing results
GCAGCCC TCACGCGTTCGAGGAGCTTGGCCCATTGCATACGTTGTATC CATAAGAATGCGGCCGCTACCGTTCGTATAATGTATGCTATACGAAGTTATGGG
5'HA
lox66
3' Junction PCR Sanger sequencing results
GCTGGGGATGCGGTGGGCTCTATGGCTTCTGataacttcgtataaagtatcctatacgaagttatACCGGTATGCATCCTGCAGGGGCTACAGTATA
GCTGGGGATGCGGTGGGCTCTATGGCTTCTGATAACTTCGTATAAAGTATCCTATACGAAGTTATACCGGTATGCATCCTGCAGGGGCTACAGTATA
lox2272
3'HA

Figure S6

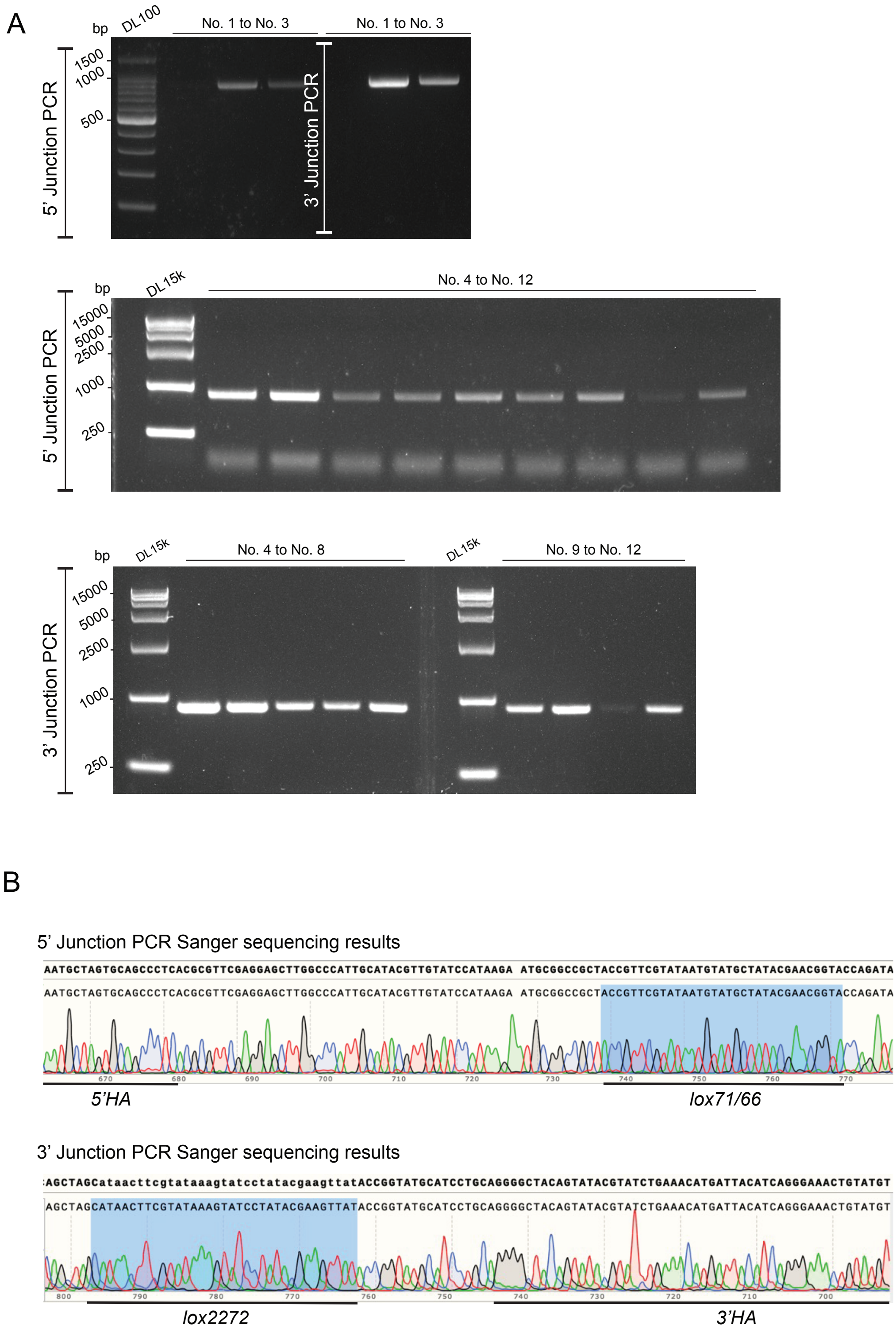
A
bp
DL100
No. 1 to No. 3
No. 1 to No. 3
1500
1000
500
5' Junction PCR
3' Junction PCR
DL15k
No. 4 to No. 12
15000
5000
2500
1000
250
5' Junction PCR
DL15k
No. 4 to No. 8
DL15k
No. 9 to No. 12
3' Junction PCR
B
5' Junction PCR Sanger sequencing results
AATGCTAGTGCAGCCCTCACGCGTTCGAGGAGCTTGGCCCATTGCATACGTTGTATCCATAAGA ATGCGGCCGCTACCGTTCGTATAATGTATGCTATACGAACGGTACCAGATA
5'HA
lox71/66
3' Junction PCR Sanger sequencing results
AGCTAGCataacttcgtataaagtatcctatacgaagttatACCGGTATGCATCCTGCAGGGGCTACAGTATACGTATCTGAAACATGATTACATCAGGGAAACTGTATGT
lox2272
3'HA

Figure S7

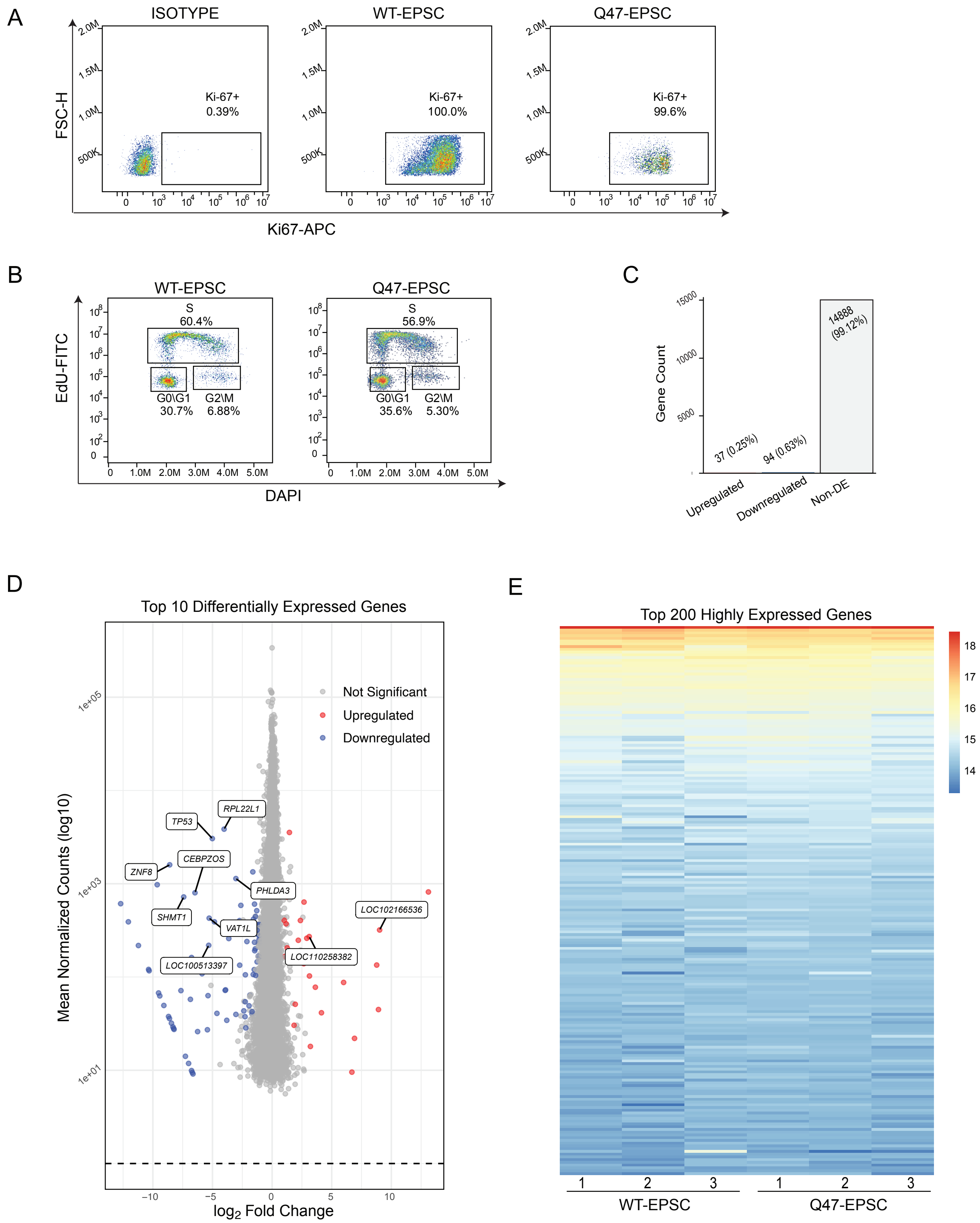
A
ISOTYPE
WT-EPSC
Q47-EPSC
FSC-H
2.0M
1.5M
1.0M
500K
Ki-67+
0.39%
Ki-67+
100.0%
Ki-67+
99.6%
Ki67-APC
B
WT-EPSC
Q47-EPSC
EdU-FITC
S
60.4%
G0\G1
30.7%
G2\M
6.88%
S
56.9%
G0\G1
35.6%
G2\M
5.30%
1.0M 2.0M 3.0M 4.0M 5.0M
DAPI
C
Gene Count
15000
10000
5000
37 (0.25%)
94 (0.63%)
14888 (99.12%)
Upregulated
Downregulated
Non-DE
D
Top 10 Differentially Expressed Genes
Not Significant
Upregulated
Downregulated
Mean Normalized Counts (log10)
1e+05
1e+03
1e+01
RPL22L1
TP53
CEBPZOS
ZNF8
PHLDA3
SHMT1
VAT1L
LOC100513397
LOC102166536
LOC110258382
−10
−5
0
5
10
log2 Fold Change
E
Top 200 Highly Expressed Genes
18
17
16
15
14
1 2 3 1 2 3
WT-EPSC
Q47-EPSC

Figure S8

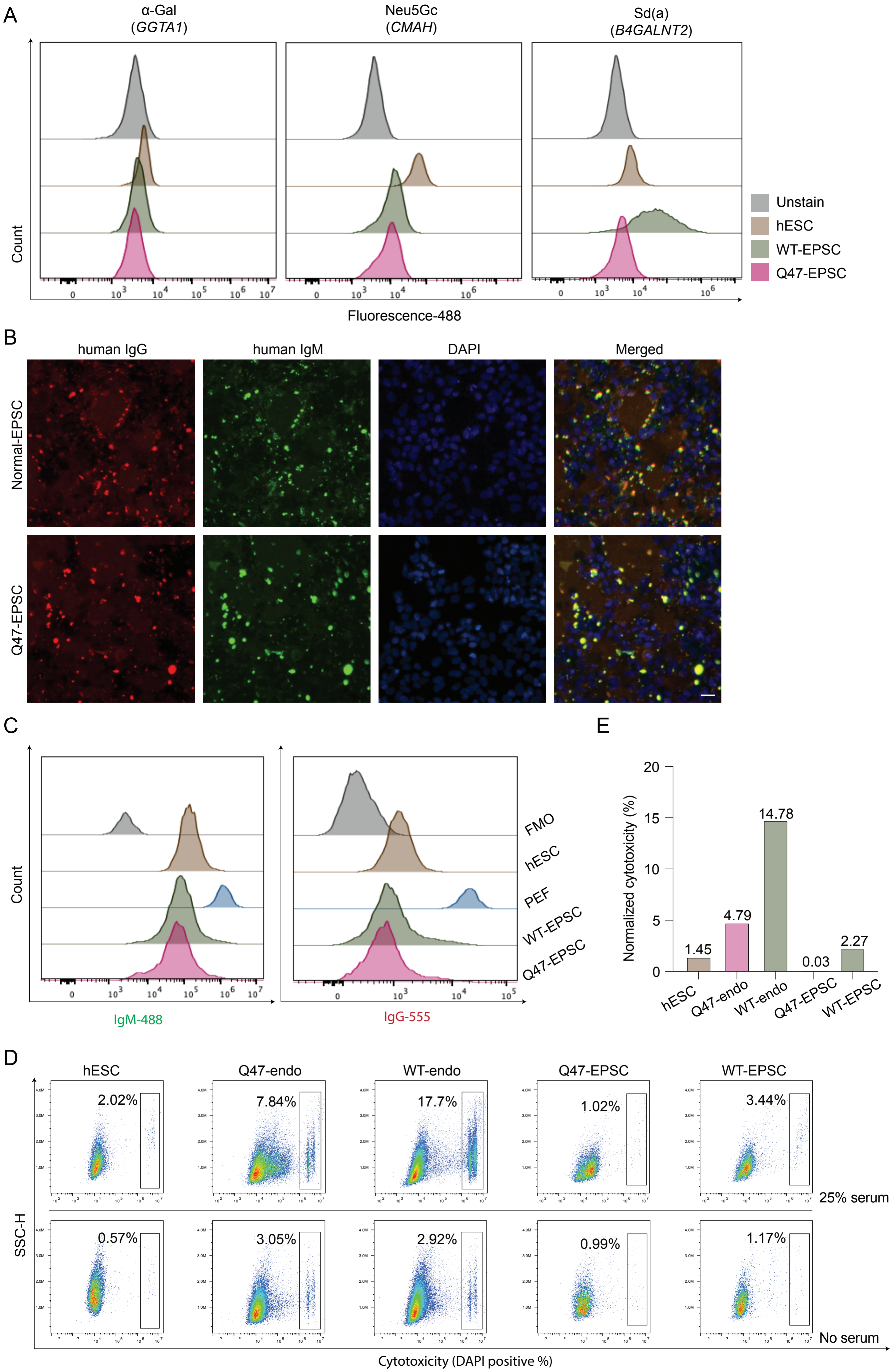
A
α-Gal
(GGTA1)
Neu5Gc
(CMAH)
Sd(a)
(B4GALNT2)
Count
Fluorescence-488
Unstain
hESC
WT-EPSC
Q47-EPSC
B
human IgG
human IgM
DAPI
Merged
Normal-EPSC
Q47-EPSC
C
Count
IgM-488
IgG-555
FMO
hESC
PEF
WT-EPSC
Q47-EPSC
E
Normalized cytotoxicity (%)
1.45
4.79
14.78
0.03
2.27
hESC
Q47-endo
WT-endo
Q47-EPSC
WT-EPSC
D
hESC
Q47-endo
WT-endo
Q47-EPSC
WT-EPSC
2.02%
7.84%
17.7%
1.02%
3.44%
25% serum
0.57%
3.05%
2.92%
0.99%
1.17%
No serum
SSC-H
Cytotoxicity (DAPI positive %)

Figure S9

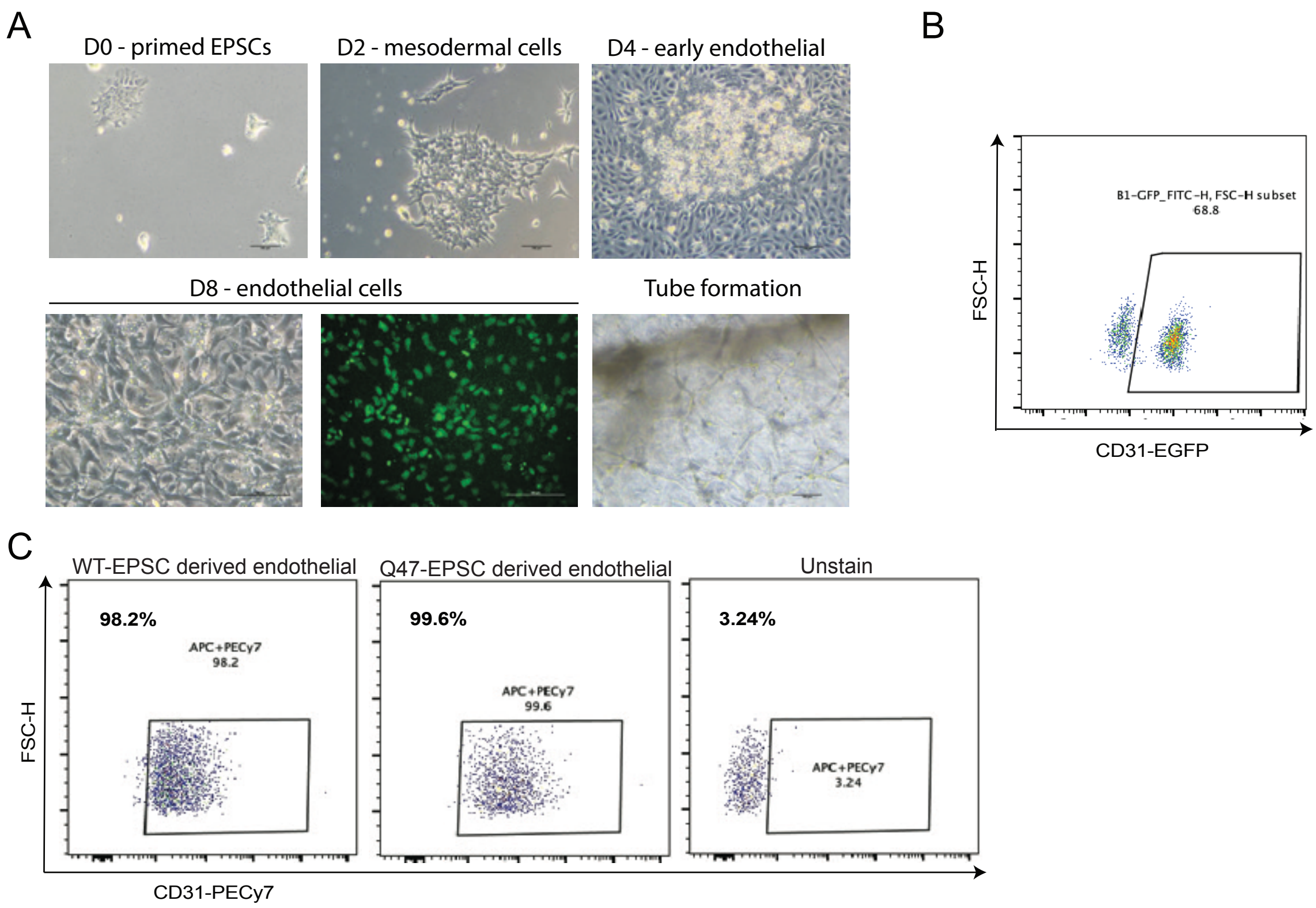
A
D0 - primed EPSCs
D2 - mesodermal cells
D4 - early endothelial
D8 - endothelial cells
Tube formation
B
B1-GFP_FITC-H, FSC-H subset
68.8
FSC-H
CD31-EGFP
C
WT-EPSC derived endothelial
Q47-EPSC derived endothelial
Unstain
98.2%
APC+PECy7
98.2
99.6%
APC+PECy7
99.6
3.24%
APC+PECy7
3.24
FSC-H
CD31-PECy7

Figure S10

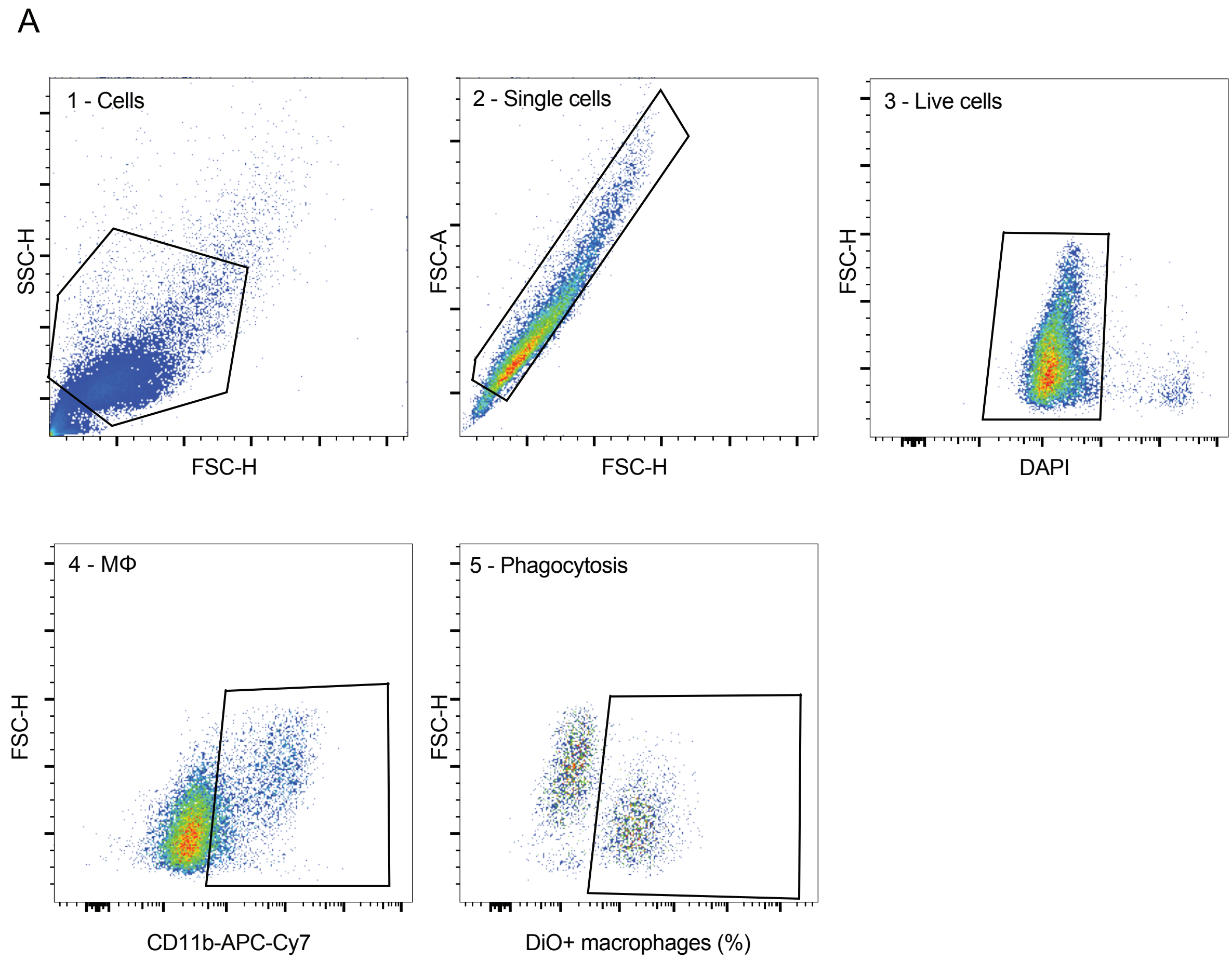
A
1 - Cells
SSC-H
FSC-H
2 - Single cells
FSC-A
FSC-H
3 - Live cells
FSC-H
DAPI
4 - MΦ
FSC-H
CD11b-APC-Cy7
5 - Phagocytosis
FSC-H
DiO+ macrophages (%)

Figure S11

A

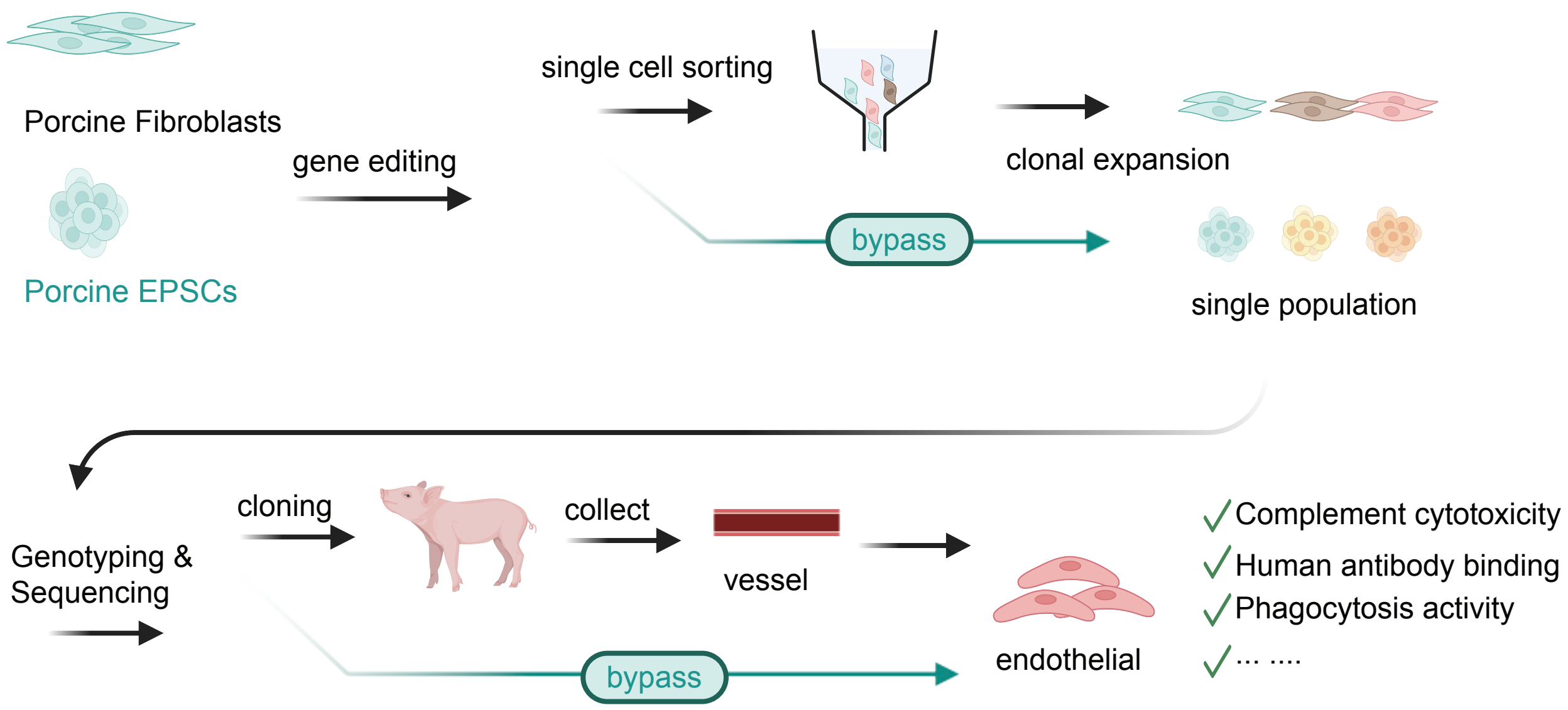
Porcine Fibroblasts
Porcine EPSCs
gene editing
single cell sorting
clonal expansion
bypass
single population
Genotyping &
Sequencing
cloning
collect
vessel
bypass
endothelial
Complement cytotoxicity
Human antibody binding
Phagocytosis activity
··· ····
