# Supplementary material for: Porcine Expanded Potential Stem Cells as a Versatile Platform for Multiplex Genome Editing and Immunophenotyping in Xenotransplantation
Source: Xenotransplantation. 2026 Aug 21;33(4):e70161. doi: 10.1111/xen.70161 (PMC13494693; doi:10.1111/xen.70161)
Supplement: Supplementary file 2 [file XEN-33-e70161-s002.docx]

**Supporting Information for**

Porcine Expanded Potential Stem Cells as a Versatile Platform for Multiplex Genome Editing and Immunophenotyping in Xenotransplantation.

Yiyi Xuan^1, 2^, Yong Xiang^3^, Xining Wang^1, 2^, Björn Petersen^4^, Pengtao Liu^1, 2, 5*^

^1^ InnoHK Centre for Translational Stem Cell Biology, Building 17W, Science Park, Hong Kong Special Administrative Region, China

^2^ Stem Cell & Regenerative Medicine Consortium, School of Biomedical Sciences, Li Ka Shing Faculty of Medicine, The University of Hong Kong, Hong Kong Special Administrative Region, China

^3^ School of Biomedical Sciences, The Chinese University of Hong Kong, Shatin, Hong Kong

^4^ FLI, Institute of Farm Animal Genetics, Mariensee, Germany

^5^ Shenzhen Key Laboratory of Fertility Regulation, Reproductive Medicine Center, The

University of Hong Kong - Shenzhen Hospital, Shenzhen, China.

***Corresponding author(s).** Pengtao Liu*

**Email:** [pliu88@hku.hk](mailto:pliu88@hku.hk).

**This file includes:**

Figure Legends S1 to S11

Tables S1 to S7

Supporting Information Text

Figures

**Fig. S1. Genetic editing strategy for quadruple-knockout (QKO) pEPSC generation.** (**A)** *GGTA1* targeting: Dual sgRNAs flanking exon 7 were employed to generate a large deletion encompassing the C-terminal catalytic domain. **(B)** *CMAH* targeting: Exon 1 was disrupted using CRISPR/Cas9 to inactivate cytidine monophosphate-N-acetylneuraminic acid hydroxylase, preventing Neu5Gc synthesis**. (C)** *B4GALNT2* targeting: Exon 3 was edited to disrupt β-1,4-N-acetylgalactosaminyltransferase function, thereby removing the Sd(a) glycan epitope. **(D)** *GHR* targeting: two sgRNAs were designed in exon 7 to induce a 110-bp deletion, to impair the function the extracellular domain of growth hormone receptor. **(A, B, C & D)** Sanger sequencing validate each genetic modification.

**Fig. S2.** **Genotyping of genome-edited pEPSCs.** Gel electrophoresis detects band shifts of editing *GGTA1* (**A)**, *CMAH* **(B)**, and *B4GALNT2* **(C)** in total 46 colonies, for generating triple knockout (TKO) pEPSCs. **(D)** Using TKO-pEPSCs for *GHR* knockout, 16 colonies were analysed for loss of the wild type PCR fragment. Primer pairs were in Table S2.

**Fig. S3.** **Genomic organization and experimental validation of the *B4GALNT2* and *B4GALNT2-like* loci.** (**A)** Schematic representation of the genomic region harboring B4GALNT2 and its paralog *B4GALNT2-like* on chromosome 12. Four intervening genes (LOC110255205, LOC102158609, LOC110255211, and TRANQ-UUG) are located between these two genes. **(B)** Summary table of the four intervening genes, including their gene abbreviations and functional descriptions. **(C)** PCR amplification of four intervening genes in the TKO cells. Gel electrophoresis confirms the presence of all four genes in the genome, supporting the annotated genomic organization. Primer pairs are listed in Table S2.

**Fig. S4. Human CD47 (hCD47) cDNA integration into the porcine *ROSA26* locus via recombinase-mediated cassette exchange (RMCE)*.*** (**A)** Schematic illustration of RMCE-mediated cassette integration at the porcine *ROSA26* locus. The RMCE donor, which includes two homologous arms and the *puroΔTK* fragment flanked by two mutant *lox66/lox2272* sites, was introduced at the *ROSA26* locus via gene targeting facilitated by the CRISPR/Cas9. The *Puromycin* resistance was used for positive selection of RMCE cassette integration into the *ROSA26* locus. In the 2^nd^ step, the *hCD47* donor cDNA was introduced into the RMCE master cells through co-electroporation with a Cre-expressing plasmid (*Cre-mCherry*), resulting in the replacement of *puroΔTK* (flanked by *lox66/lox2272*) with *hCD47* (flanked by *loxP/Lox2272*). The *ΔTK* cassette was employed to negatively select using *ganciclovir* against cells that still retain this cassette after RMCE. **(B)** The genotyping information for RMCE insertion into the *ROSA26* locus and for *hCD47* replacing the RMCE cassette.

**Fig. S5.** **Genotyping results of constructing** **the RMCE master cell line.** (**A)** Gel electrophoresis of 5’ and 3’ junction fragment genotyping of RMCE cassette integration at the *ROSA26* locus. **(B)** Sanger sequencing results confirm the RMCE integration. Primer pairs were in Table S2.

**Fig. S6.** **Human CD47 (hCD47) insertion by Cre-*loxP* recombination via cassette exchange.** (**A)** Gel electrophoresis detects the expected 5’ and 3’ junction fragments for *hCD47* integration into the *ROSA26* locus. **(B)** Sanger sequencing confirms the *hCD47* integration. Primer pairs were in Table S2.

**Fig. S7.** **Molecular characterization of QKO with human CD47 (Q47) integrated pEPSCs (Q47-pEPSC).** (**A, B)** Proliferation analysis revealed 99.6% were proliferative (Ki-67 positive, A) and 56.9% of Q47-pEPSC were in S-phase (EdU assay, B). **(C)** Summary of differentially expressed genes (DEGs) (adjusted P < 0.05, |log2FC| > 1) between normal or wildtype (WT) and Q47 pEPSCs. The cell lines share 99.12% transcriptional similarity. **(D)** MA plot showing differentially expressed genes (DEGs) between WT- and Q47-pEPSCs. Red and blue dots represent significantly upregulated and downregulated genes (|log2FC| > 1, padj < 0.05), respectively. Gray dots indicate non-significant genes. Dashed horizontal lines mark |log2FC| = 1 threshold. Selected top DEGs are labeled. **(E)** Heatmap of top 200 highly expressed genes. (C, D, E) Data were from bulk RNA-seq. Q47 refers to quadruple knockout (QKO, *GGTA1-/- CMAH-/- B4GALNT2-/- GHR-/-)* with human *CD47* cDNA integration.

**Fig. S8.** **Immunophenotyping porcine EPSCs.** (**A)** Flow cytometry analysis of α-Gal (*GGTA1*), Neu5Gc (*CMAH*), and Sd(a) (*B4GALNT2*) on Q47 and wildtype (WT) pEPSCs. Unstained pEPSCs and human embryonic stem cells (hESC) were used as the negative controls. **(B)** Immunofluorescence staining of human IgG (red) and IgM (green) binding on Q47 and WT pEPSCs. Nuclei were counterstained with DAPI (blue). Scale bars: 25 µm. **(C)** Flow cytometry analysis of human IgG and IgM binding on Q47 and WT pEPSCs. FMO (fluorescence minus one control) and hESCs were used as the negative controls. Porcine embryonic fibroblast (PEF) were used as the positive control. **(D)** Representative flow cytometry dot plots of the complement-mediated cytotoxicity assay. DAPI+ (dead) cells were analyzed after incubation with heat-inactivated human serum plus complement in different cell types (hESC, Q47-Endo, WT-Endo, Q47-pEPSC, and WT-pEPSC). **(E)** Quantification of normalized cytotoxicity (percentage of DAPI+ cells in the serum-treated group minus the no-serum control) across cell types. Data are from a single representative experiment.

**Fig. S9.** **Generation of endothelial cells from pEPSCs.** (**A)** Brightfield images illustrating the time course of endothelial differentiation from pEPSCs and tube formation assay. **(B)** Flow cytometry analysis detects endothelial cells (GFP^+^) differentiated from porcine CD31-EGFP EPSC reporter cell line. **(C)** Flow cytometry analysis detects endothelial cells differentiated from Q47 and WT pEPSCs. Q47-endo refers to Q47-pEPSC derived endothelial cells, WT-endo refers to WT-pEPSC derived endothelial cells. Q47 refers to quadruple knockout (QKO, *GGTA1-/- CMAH-/- B4GALNT2-/- GHR-/-)* with human *CD47* cDNA integration.

**Fig. S10. Gating strategy for analysing phagocytosis.** (**A)** Firstly, forward and side scatter gating is performed to select the cells of interest. Next, single cells using A and H parameters under forward and side scatter are selected. Then gate DAPI negative cells were identified as live cells; gate CD11b positive macrophages in the live cell population. Finally, the subpopulation that has phagocytosed the DiOc labeled target cells was further gated as phagocytosis cells.

**Fig. S11. Comparison between pEPSC- and fibroblast-based system relevant to xenotransplantation.** (**A)** In conventional fibroblast-based systems, genetic modification requires subsequent single-cell sorting to isolate edited clones. pEPSCs, however, naturally form single-cell colonies after electroporation, bypassing post-editing sorting. Additionally, pEPSCs permit direct endothelial differentiation for functional assessment of edits, circumventing the animal cloning and tissue harvesting steps needed with fibroblasts. This streamlined workflow enables faster evaluation of editing outcomes.

Tables

| **Table S1. Summary of guide RNA sequences** | | |
| --- | --- | --- |
| **Name** | **Sequence (5' to 3')** | **Format** |
| GGTA1-g1 | UUAGGAUUGUCAAGAUAGCU | synthetic sgRNA |
| GGTA1-g2 | GAUGCGCAUGAAGACCAUCG | synthetic sgRNA |
| CMAH-g1 | GUUUGUUCGAUGCUAUGAAA | synthetic sgRNA |
| CMAH-g2 | UAAGAAUAAGAGCCGCCUGA | synthetic sgRNA |
| B4GALNT2-g1 | UCCUCAGGUUCACUGCGGGG | synthetic sgRNA |
| B4GALNT2-g2 | AUGUGACGCCUUCGGGCAUC | synthetic sgRNA |
| GHR-g1 | GUGCAACCGGAUCCACCCAU | synthetic sgRNA |
| GHR-g2 | UCAGAAGGGAUGGAUAGUCC | synthetic sgRNA |
| pROSA26 | CAAUGCUAGUGCAGCCCUCA | synthetic sgRNA |

| **Table S2. Summary of genotyping primers** | | |
| --- | --- | --- |
| **Name** | **Sequence (5' to 3')** | **purpose** |
| GGTA1-GTP4 | CATGGTTGGCCACAAAGTCATC | GGTA1 KO |
| GGTA1-GTP6 | CCACAGAGTCATTCTCCCATTTGAC |  |
| CMAH- GTP1 | GCTGTCAATGCTCAGGGATTCTC | CMAH KO |
| CMAH- GTP2 | GCAAAGGATTCTCAGTACCTTCC |  |
| B4GAL- GTP1 | CCCTCACCTACCAGCCCACT | B4GALNT2 KO |
| B4GAL- GTP2 | AGCTTCCGCTCCATCTCAGG |  |
| GHR-GTP1 | CTGTTGCCAACATCCTGTTGTAGG | GHR KO |
| GHR-GTP4 | ATTGTAATGGGGAGGTTCTGG |  |
| TK-6 | CCATGCACGTCTTTATCCTG | pROSA26 KI, 3' junction |
| pROSA-KIGT- 3R | GAGGAAGTATAGCACAGGAAGAGGATAAGTG |  |
| pROSA-KIGT- 5F | GGCTGTAGCCACAAATTTGTGC | pROSA26 KI, 5' junction |
| TK-1 | GTCCCATAAGGTCATGTACTGG |  |
| pROSA-KIGT- 5F | GGCTGTAGCCACAAATTTGTGC | hCD47 integration at pROSA26, 5' junction |
| pROSA-hCD47- 5R1 | GGAGATCAACGCGTATATCTGG |  |
| pROSA-hCD47- 3F1 | GTCGTATCGATCCTCAGCTAGC | hCD47 integration at pROSA26, 3' junction |
| pROSA-KIGT- 3R | GAGGAAGTATAGCACAGGAAGAGGATAAGTG |  |
| Phos-F | TGTCTGCGTCATGTGTAGTG | PHOS amplification |
| Phos-R | GTAGGAGCGGTCCAAGAAATAG |  |
| Guan-F | GGCGGTGTGTGAGAGTATTT | Guan amplification |
| Guan-R | GGACCTATCTATGGCGGAAATG |  |
| ABI-F | CTCATGGTTCCTAGCTGGATTT | ABI amplification |
| ABI-R | CTGTCTCTCTGTCTGTCTCTCT |  |
| TRNA-F | GGAACCATCCATCCACCAAATA | TRNA amplification |
| TRNA-R | GAGCCTTCTCAAGCCACATAG |  |

| **Table S3. Summary of qPCR primers** | |
| --- | --- |
| **Name** | **sequence (5' to 3')** |
| Q-pGAPDH-F | ACCCAGAAGACTGTGGATGG |
| Q-pGAPDH-R | ACGCCTGCTTCACCACCTTC |
| Q-pOCT4-F | TGTACCAGGGCTTTGGGATTAAGTTC |
| Q-pOCT4-R | ATTGAACTTCACCTTCCCTCCAACC |
| Q-pSOX2-F | GTGAGGGCCGGACAGTGAACTG |
| Q-pSOX2-R | AAGCGTACCGGGTTTTTCTCCATAC |
| Q-pNANOG-F | CTGGAGGAGATCTTCATGATTCTAAG |
| Q-pNANOG-R | AGTCCAGGCTTAAGTGTCTAGATAGAAG |
| Q-pT-F | GCAAAGTCAAGCTCACCAAC |
| Q-pT-R | TGAACTGGGTCTCTGGGAAG |
| Q-pPAX6-F | CCGAATTCTGCAGACCCATG |
| Q-pPAX6-R | TACTACCACCGATTGCCCTG |
| Q-pGATA2-F | CTCCAGCTTCACCCCTAAG |
| Q-pGATA2-R | CCCGTTCATCTTGTGGTACAG |
| Q-pFOXA2-F | CCA ACA AGA TGC TGA CCC TG |
| Q-pFOXA2-R | AGG AAG CAG TCG TTG AAG GA |
| q-GGTA1-F | CCCAGCTTCTGCCGATCA |
| q-GGTA1-R | ACTGCTGCCAACTTCTGGGT |
| Q-CMAH-F | AGGTGGTAACCTGGACCCCA |
| Q-CMAH-R | TGGAGGCTCTACGATGCCCT |
| Q-B4GALNT2-F | GAGGCGGCGGGATTAAGGAT |
| Q-B4GALNT2-R | TAGAAGCTTCAGCGTCTGCG |
| Q-GHR-F | CCACACCAGCTGTCCTTGTCA |
| Q-GHR-R | ACGGACCCCATCTGTCCAGT |

| **Table S4. Summary of antibodies** | | | |
| --- | --- | --- | --- |
| **Antibodies** | **Catalogue** | **Purposes** | **Dilution** |
| Oct3/4 (C10) | Santa Cruz, sc-5279 | IF | 1:200 |
| SOX2 | R&D, AF2018 | IF | 1:200 |
| NANOG | Peprotech, 500-P236 | IF | 1:250 |
| Tubulin β-III-594 | abcam, AB201740 | IF | 1:100 |
| AFP-488 | abcam, AB199231 | IF | 1:100 |
| SMA-488 | abcam, AB223920 | IF | 1:100 |
| GAPDH | invitrogen, AM4300 | WB | 1:5000 |
| GHR | R&D, AF1210 | WB | 1:1000 |
| CD47 (B6H12) | invitrogen, 14-0479-82 | IF | 1:200 |
| CD11b (ICRF44) -APC | invitrogen, 17-0118-42 | FLow | 1:100 |
| Mouse anti Pig CD31:APC | Bio-Rad, MCA1746APC | Flow | 1:10 |
| VE-Cadherin | CST, 2500 | IF | 1:100 |
| Ki-67-APC | BioLegend, 350513 | Flow | 1:20 |
| Isolectin B4, DyLightTM 594 | Vector Laboratories, DL-1207-.5 | IF | 1:100 |
| Isolectin B4, FITC | Sigma-Aldrich, L2895 | Flow | 1:100 |
| DBA, FITC | invitrogen, L32474 | IF, Flow | 1:100 |
| Neu5Gc kit | BioLegend, 146901 | IF, Flow | 1:100 |
| Goat anti-Chicken IgY (H+L) | invitrogen, A-11039 | IF, Flow | 1:500 |
| Rabbit anti Goat IgG (H+L), HRP | Invitrogen, 31402 | WB | 1:5000 |
| Goat anti Mouse IgG (H+L) , HRP | Invitrogen, 31430 | WB | 1:5000 |
| Goat anti-Human IgG, AF 555 | invitrogen, A-21433 | IF, Flow | 1:200 |
| Goat anti-Human IgM, AF 488 | invitrogen, A-21215 | IF, Flow | 1:200 |
| Donkey-anti-Mouse IgG, AF 488 | invitrogen, A21202 | IF | 1:1000 |
| Donkey-anti-Goat IgG , AF 594 | invitrogen, A32758 | IF | 1:1000 |
| Donkey-anti-Rabbit IgG, AF647 | invitrogen, A31573 | IF | 1:1000 |

| **Table S5. Summary of karyotyping** | | | |
| --- | --- | --- | --- |
| Mitosis phases | | Numbers (%) | |
|  |  | WT | Q47 |
| Normal | 38 | 16 (89.89%) | 18 (85.714%) |
| Abnormal | 37 | 2 (11.11%) | 1 (4.762%) |
|  | 39 | 0 (0%) | 2 (9.5245%) |
| Total | | 18 | 21 |

| **Table S6. Summary of off-target analysis** | | | | | | | |
| --- | --- | --- | --- | --- | --- | --- | --- |
| **sgRNA** | **Predicted sites** | **0 mismatches** | **1 mismatch** | **2 mismatches** | **3 mismatches** | **Confirmed off-target by WGS** | |
|  |  |  |  |  |  | INDEL/SNP off-target | SV  off-target |
| GGTA1-g1 | 158 | 1 | 0 | 1 | 156 | 0 | 0 |
| GGTA1-g2 | 24 | 1 | 0 | 0 | 23 | 0 | 0 |
| CMAH-g1 | 140 | 1 | 0 | 0 | 139 | 0 | 0 |
| CMAH-g2 | 85 | 1 | 0 | 0 | 84 | 0 | 0 |
| B4GALNT2-g1 | 147 | 2 | 0 | 2 | 143 | 0 | 0 |
| B4GALNT2-g2 | 28 | 2 | 0 | 0 | 26 | 0 | 0 |
| GHR-g1 | 32 | 0 | 0 | 0 | 32 | 0 | 0 |
| GHR-g2 | 115 | 0 | 1 | 2 | 112 | 0 | 0 |
| pROSA26 | 184 | 0 | 1 | 25 | 158 | 0 | 0 |
| **TOTAL** | **913** | **8** | **2** | **30** | **873** | **0** | **0** |
| Abbreviations: INDEL, insertion/deletion; SV, structural variant; WGS, whole-genome sequencing. | | | | | | | |

**Table S7. Sequences of the donor cassettes used for human *CD47* integration at the porcine *ROSA26* locus**

RMCE donor vector sequence

GTAAAACGACGGCCAGTGAATTGTAATACGACTCACTATAGGGCGAATTGGAGCTCCACCGCGGTGGCGGCCGCATTGATAATTTTCTTTTCATACTAAGCAGTAAGGAAAGAAAAGTTGAAACTCTGTGGTCCATTTAGGTTATATGTGTATTTGTACTTGATTGGTTTGTTTGAATACCTATTTCTATACTTTAGCTGAGAGCTAAAGCCAACAAACCAGTACTGTAGATAACCTGCTTTGGACAACAATGTGTTGACTAGTTGGATTTCACCAAAGAATGCCTAATAAATTTTAAGAAAATGAGATTTCATTAAACCATAATACTGACATAAGTTTAGGGAAGAATCAGACTATATCTGGTGTTTGTGAAACTACCCCTGAATTTCAGTCCTACAAAGTTTTCAGTTTTGGAAAAACTTTCATCAGAGAGGGCACTAAGTTACAGGAAGCCATCACAAAGTAAGTTTTCATCTGATGAATTATAAATTTAAGATATATTTTAATACCAAAATTCTTTATGGTTTATGTGCTAACTTAAAATTTCTCCTTAAAATATGAGAACTAAGTACACAATTGTACTTGGCTGTTTAATGCGGATTCCCAGTCCCTCACACAGAGATTCTCAATTAAGATTGGAGAGCAGGGGTTACTAGAATTCTTTTTCAGGTTCCTTATATGCTTCTGATTTGGTGGCCTAGAAATCACAATGCTAGTGCAGCCCTCACGCGTTCGAGGAGCTTGGCCCATTGCATACGTTGTATCCATAAGAATGCGGCCGCtaccgttcgtataatgtatgctatacgaagttatGGGTCGACATTGATTATTGACTAGTTATTAATAGTAATCAATTACGGGGTCATTAGTTCATAGCCCATATATGGAGTTCCGCGTTACATAACTTACGGTAAATGGCCCGCCTGGCTGACCGCCCAACGACCCCCGCCCATTGACGTCAATAATGACGTATGTTCCCATAGTAACGCCAATAGGGACTTTCCATTGACGTCAATGGGTGGACTATTTACGGTAAACTGCCCACTTGGCAGTACATCAAGTGTATCATATGCCAAGTACGCCCCCTATTGACGTCAATGACGGTAAATGGCCCGCCTGGCATTATGCCCAGTACATGACCTTATGGGACTTTCCTACTTGGCAGTACATCTACGTATTAGTCATCGCTATTACCATGGGTCGAGGTGAGCCCCACGTTCTGCTTCACTCTCCCCATCTCCCCCCCCTCCCCACCCCCAATTTTGTATTTATTTATTTTTTAATTATTTTGTGCAGCGATGGGGGCGGGGGGGGGGGGGGCGCGCGCCAGGCGGGGCGGGGCGGGGCGAGGGGCGGGGCGGGGCGAGGCGGAGAGGTGCGGCGGCAGCCAATCAGAGCGGCGCGCTCCGAAAGTTTCCTTTTATGGCGAGGCGGCGGCGGCGGCGGCCCTATAAAAAGCGAAGCGCGCGGCGGGCGGGAGTCGCTGCGTTGCCTTCGCCCCGTGCCCCGCTCCGCGCCGCCTCGCGCCGCCCGCCCCGGCTCTGACTGACCGCGTTACTCCCACAGGTGAGCGGGCGGGACGGCCCTTCTCCTCCGGGCTGTAATTAGCGCTTGGTTTAATGACGGCTCGTTTCTTTTCTGTGGCTGCGTGAAAGCCTTAAAGGGCTCCGGGAGGGCCCTTTGTGCGGGGGGGAGCGGCTCGGGGGGTGCGTGCGTGTGTGTGTGCGTGGGGAGCGCCGCGTGCGGCCCGCGCTGCCCGGCGGCTGTGAGCGCTGCGGGCGCGGCGCGGGGCTTTGTGCGCTCCGCGTGTGCGCGAGGGGAGCGCGGCCGGGGGCGGTGCCCCGCGGTGCGGGGGGGCTGCGAGGGGAACAAAGGCTGCGTGCGGGGTGTGTGCGTGGGGGGGTGAGCAGGGGGTGTGGGCGCGGCGGTCGGGCTGTAACCCCCCCCTGCACCCCCCTCCCCGAGTTGCTGAGCACGGCCCGGCTTCGGGTGCGGGGCTCCGTGCGGGGCGTGGCGCGGGGCTCGCCGTGCCGGGCGGGGGGTGGCGGCAGGTGGGGGTGCCGGGCGGGGCGGGGCCGCCTCGGGCCGGGGAGGGCTCGGGGGAGGGGCGCGGCGGCCCCGGAGCGCCGGCGGCTGTCGAGGCGCGGCGAGCCGCAGCCATTGCCTTTTATGGTAATCGTGCGAGAGGGCGCAGGGACTTCCTTTGTCCCAAATCTGGCGGAGCCGAAATCTGGGAGGCGCCGCCGCACCCCCTCTAGCGGGCGCGGGCGAAGCGGTGCGGCGCCGGCAGGAAGGAAATGGGCGGGGAGGGCCTTCGTGCGTCGCCGCGCCGCCGTCCCCTTCTCCATCTCCAGCCTCGGGGCTGCCGCAGGGGGACGGCTGCCTTCGGGGGGGACGGGGCAGGGCGGGGTTCGGCTTCTGGCGTGTGACCGGCGGCTCTAGAGCCTCTGCTAACCATGTTCATGCCTTCTTCTTTTTCCTACAGCTCCTGGGCAACGTGCTGGTTGTTGTGCTGTCTCATCATTTTGGCAAAGAATTGGGATCCGGTACCgagacgACGCGTATGGGGACCGAGTACAAGCCCACGGTGCGCCTCGCCACCCGCGACGACGTCCCCCGGGCCGTACGCACCCTCGCCGCCGCGTTCGCCGACTACCCCGCCACGCGCCACACCGTCGACCCGGACCGCCACATCGAGCGGGTCACCGAGCTGCAAGAACTCTTCCTCACGCGCGTCGGGCTCGACATCGGCAAGGTGTGGGTCGCGGACGACGGCGCCGCGGTGGCGGTCTGGACCACGCCGGAGAGCGTCGAAGCGGGGGCGGTGTTCGCCGAGATCGGCCCGCGCATGGCCGAGTTGAGCGGTTCCCGGCTGGCCGCGCAGCAACAGATGGAAGGCCTCCTGGCGCCGCACCGGCCCAAGGAGCCCGCGTGGTTCCTGGCCACCGTCGGCGTCTCGCCCGACCACCAGGGCAAGGGTCTGGGCAGCGCCGTCGTGCTCCCCGGAGTGGAGGCGGCCGAGCGCGCCGGGGTGCCCGCCTTCCTGGAGACCTCCGCGCCCCGCAACCTCCCCTTCTACGAGCGGCTCGGCTTCACCGTCACCGCCGACGTCGAGGTGCCCGAAGGACCGCGCACCTGGTGCATGACCCGCAAGCCCGGTGCCGGATCCATGCCCACGCTACTGCGGGTTTATATAGACGGTCCTCACGGGATGGGGAAAACCACCACCACGCAACTGCTGGTGGCCCTGGGTTCGCGCGACGATATCGTCTACGTACCCGAGCCGATGACTTACTGGCAGGTGCTGGGGGCTTCCGAGACAATCGCGAACATCTACACCACACAACACCGCCTCGACCAGGGTGAGATATCGGCCGGGGACGCGGCGGTGGTAATGACAAGCGCCCAGATAACAATGGGCATGCCTTATGCCGTGACCGACGCCGTTCTGGCTCCTCATATCGGGGGGGAGGCTGGGAGCTCACATGCCCCGCCCCCGGCCCTCACCCTCATCTTCGACCGCCATCCCATCGCCGCCCTCCTGTGCTACCCGGCCGCGCGATACCTTATGGGCAGCATGACCCCCCAGGCCGTGCTGGCGTTCGTGGCCCTCATCCCGCCGACCTTGCCCGGCACAAACATCGTGTTGGGGGCCCTTCCGGAGGACAGACACATCGACCGCCTGGCCAAACGCCAGCGCCCCGGCGAGCGGCTTGACCTGGCTATGCTGGCCGCGATTCGCCGCGTTTACGGGCTGCTTGCCAATACGGTGCGGTATCTGCAGGGCGGCGGGTCGTGGCGGGAGGATTGGGGACAGCTTTCGGGGACGGCCGTGCCGCCCCAGGGTGCCGAGCCCCAGAGCAACGCGGGCCCACGACCCCATATCGGGGACACGTTATTTACCCTGTTTCGGGCCCCCGAGTTGCTGGCCCCCAACGGCGACCTGTACAACGTGTTTGCCTGGGCCTTGGACGTCTTGGCCAAACGCCTCCGTCCCATGCACGTCTTTATCCTGGATTACGACCAATCGCCCGCCGGCTGCCGGGACGCCCTGCTGCAACTTACCTCCGGGATGGTCCAGACCCACGTCACCACCCCCGGCTCCATACCGACGATCTGCGACCTGGCGCGCACGTTTGCCCGGGAGATGGGGGAGGCTAACTGAGAATTCTGCAGCTGGTGGAAGGTGTCCAAGAGCAGGTGAATGCCGCCCTGCTGGACTACACGGTTTGCAACTACCCACAACAGACTGAGAAATTCGGACAGCTACTTCTTCGGCTACCCGAGATCCGGGCAATCAGCAAGCAGGCAGAAGACTACCTGTACTATAAGCACGTGAACGGGGATGTGCCCTATAATAACCTCCTCATTGAGATGCTGCATGCCAAAAGAGCCTAATCTAGAGCTCGCTGATCAGCCTCGACTGTGCCTTCTAGTTGCCAGCCATCTGTTGTTTGCCCCTCCCCCGTGCCTTCCTTGACCCTGGAAGGTGCCACTCCCACTGTCCTTTCCTAATAAAATGAGGAAATTGCATCGCATTGTCTGAGTAGGTGTCATTCTATTCTGGGGGGTGGGGTGGGGCAGGACAGCAAGGGGGAGGATTGGGAAGACAATAGCAGGCATGCTGGGGATGCGGTGGGCTCTATGGCTTCTGataacttcgtataaagtatcctatacgaagttatACCGGTATGCATCCTGCAGGGGCTACAGTATACGTATCTGAAACATGATTACATCAGGGAAACTGTATGTCTAATCTACTTTGTCCCTAAAGGAAGCATTTTGAAAGGCAGAAAGTAATATGTGATAGTTTTTGAAACTTGTAGGTCACATTGTTTTTAAAAGGGATCCAAGTAAGTTTTTTTTTCTTTTGAGGGCTACACCTGTGGCACATGGAGGTTCCCAGGCTAGGGGTTAAATCAGAACTGCAGCTGCCAGCCTATGCCAGAGCCACAGCAATGCCAGATCTGAGCTGTGTCTGCAACTGTGTAGCTCACAGCAACGCTGGATCCTTAACCCAATGAGCAAGGCCAGAGATTGAACCTACAACCTCGTGGTTCCTAGTTAGATTTGTTTCCGCTGTGCCACGATGGGAACTCCAAGTAATTTTTTTTTGAGCAAGGAAGTTACCTTTTTTGTCTGTTTTCCCACTAAATGCATTCCTCAAGGATTCCCAGTTTGTTCTTGATTCCTCAGTGCCTTAACACAGACCTGGGTTCTCAGTAAATGTTGATTTTATTGATTTATATGTGAAATTGTTTTTCAAATAATAGTTTTTAAGTCCATAGAAACAATGCTTCTTTTATGGAGATACTTTAGGATCATACTTGTAACCCAAGTTGCCTAATACCTCGAGGGGGGGCCCGGTACCAGCTTTTGTTCCCTTTAGTGAGGGTTAATTTCGAGCTTGGCGTAATCATGGTCATAGCTGTTTCCTGTGTGAAATTGTTATCCGCTCACAATTCCACACAACATACGAGCCGGAAGCATAAAGTGTAAAGCCTGGGGTGCCTAATGAGTGAGCTAACTCACATTAATTGCGTTGCGCTCACTGCCCGCTTTCCAGTCGGGAAACCTGTCGTGCCAGCTGCATTAATGAATCGGCCAACGCGCGGGGAGAGGCGGTTTGCGTATTGGGCGCTCTTCCGCTTCCTCGCTCACTGACTCGCTGCGCTCGGTCGTTCGGCTGCGGCGAGCGGTATCAGCTCACTCAAAGGCGGTAATACGGTTATCCACAGAATCAGGGGATAACGCAGGAAAGAACATGTGAGCAAAAGGCCAGCAAAAGGCCAGGAACCGTAAAAAGGCCGCGTTGCTGGCGTTTTTCCATAGGCTCCGCCCCCCTGACGAGCATCACAAAAATCGACGCTCAAGTCAGAGGTGGCGAAACCCGACAGGACTATAAAGATACCAGGCGTTTCCCCCTGGAAGCTCCCTCGTGCGCTCTCCTGTTCCGACCCTGCCGCTTACCGGATACCTGTCCGCCTTTCTCCCTTCGGGAAGCGTGGCGCTTTCTCATAGCTCACGCTGTAGGTATCTCAGTTCGGTGTAGGTCGTTCGCTCCAAGCTGGGCTGTGTGCACGAACCCCCCGTTCAGCCCGACCGCTGCGCCTTATCCGGTAACTATCGTCTTGAGTCCAACCCGGTAAGACACGACTTATCGCCACTGGCAGCAGCCACTGGTAACAGGATTAGCAGAGCGAGGTATGTAGGCGGTGCTACAGAGTTCTTGAAGTGGTGGCCTAACTACGGCTACACTAGAAGAACAGTATTTGGTATCTGCGCTCTGCTGAAGCCAGTTACCTTCGGAAAAAGAGTTGGTAGCTCTTGATCCGGCAAACAAACCACCGCTGGTAGCGGTGGTTTTTTTGTTTGCAAGCAGCAGATTACGCGCAGAAAAAAAGGATCTCAAGAAGATCCTTTGATCTTTTCTACGGGGTCTGACGCTCAGTGGAACGAAAACTCACGTTAAGGGATTTTGGTCATGAGATTATCAAAAAGGATCTTCACCTAGATCCTTTTAAATTAAAAATGAAGTTTTAAATCAATCTAAAGTATATATGAGTAAACTTGGTCTGACAGTTACCAATGCTTAATCAGTGAGGCACCTATCTCAGCGATCTGTCTATTTCGTTCATCCATAGTTGCCTGACTCCCCGTCGTGTAGATAACTACGATACGGGAGGGCTTACCATCTGGCCCCAGTGCTGCAATGATACCGCGAGACCCACGCTCACCGGCTCCAGATTTATCAGCAATAAACCAGCCAGCCGGAAGGGCCGAGCGCAGAAGTGGTCCTGCAACTTTATCCGCCTCCATCCAGTCTATTAATTGTTGCCGGGAAGCTAGAGTAAGTAGTTCGCCAGTTAATAGTTTGCGCAACGTTGTTGCCATTGCTACAGGCATCGTGGTGTCACGCTCGTCGTTTGGTATGGCTTCATTCAGCTCCGGTTCCCAACGATCAAGGCGAGTTACATGATCCCCCATGTTGTGCAAAAAAGCGGTTAGCTCCTTCGGTCCTCCGATCGTTGTCAGAAGTAAGTTGGCCGCAGTGTTATCACTCATGGTTATGGCAGCACTGCATAATTCTCTTACTGTCATGCCATCCGTAAGATGCTTTTCTGTGACTGGTGAGTACTCAACCAAGTCATTCTGAGAATAGTGTATGCGGCGACCGAGTTGCTCTTGCCCGGCGTCAATACGGGATAATACCGCGCCACATAGCAGAACTTTAAAAGTGCTCATCATTGGAAAACGTTCTTCGGGGCGAAAACTCTCAAGGATCTTACCGCTGTTGAGATCCAGTTCGATGTAACCCACTCGTGCACCCAACTGATCTTCAGCATCTTTTACTTTCACCAGCGTTTCTGGGTGAGCAAAAACAGGAAGGCAAAATGCCGCAAAAAAGGGAATAAGGGCGACACGGAAATGTTGAATACTCATACTCTTCCTTTTTCAATATTATTGAAGCATTTATCAGGGTTATTGTCTCATGAGCGGATACATATTTGAATGTATTTAGAAAAATAAACAAATAGGGGTTCCGCGCACATTTCCCCGAAAAGTGCCACCTCTCAAGGATCTTACCGCTGTTGAGATCCAGTTCGATGTAACCCATTCGTGCACCCAACTTGATCTTCAGCATCTTTTACTTTCACCAGCGTTTCTGGGTGAGCAAAAACAGGAAGGCAAAATGCCGCAAAAAAGGGAATAAGGGCGACACGGAAATGTTGAATACTCATACTCTTCCTTTTTCAATATTATTGAAGCATTTATCAGGGTTATTGTCTCATGAGCGGATACATATTTGAATGTATTTAGAAAAATAAACAAATAGGGGTTCCGCGCACATTTCCCCGAAAAGTGCCACCTGACGCGCCCTGTAGCGGCGCATTAAGCGCGGCGGGTGTGGTGGTTACGCGCAGCGTGACCGCTACACTTGCCAGCGCCCTAGCGCCCGCTCCTTTCGCTTTCTTCCCTTCCTTTCTCGCCACGTTCGCCGGCTTTCCCCGTCAAGCTCTAAATCGGGGGCTCCCTTTAGGGTTCCGATTTAGTGCTTTACGGCACCTCGACCCCAAAAAACTTGATTAGGGTGATGGTTCACGTAGTGGGCCATCGCCCTGATAGACGGTTTTTCGCCCTTTGACGTTGGAGTCCACGTTCTTTAATAGTGGACTCTTGTTCCAAACTGGAACAACACTCAACCCTATCTCGGTCTATTCTTTTGATTTATAAGGGATTTTGCCGATTTCGGCCTATTGGTTAAAAAATGAGCTGATTTAACAAAAATTTAACGCGAATTTTAACAAAATATTAACGCTTACAATTTCCATTCGCCATTCAGGCTGCGCAACTGTTGGGAAGGGCGATCGGTGCGGGCCTCTTCGCTATTACGCCAGCTGGCGAAAGGGGGATGTGCTGCAAGGCGATTAAGTTGGGTAACGCCAGGGTTTTCCCAGTCACGACGTT

Human *CD47* donor PCR fragment sequence

ataacttcgtataatgtatgctatacgaacggtaCCAGATATACGCGTTGATCTCCATAAGAGAAGAGGGACAGCTATGACTGGGAGTAGTCAGGAGAGGAGGAAAAATCTGGCTAGTAAAACATGTAAGGAAAATTTTAGGGATGTTAAAGAAAAAAATAACACAAAACAAAATATAAAAAAAATCTAACCTCAAGTCAAGGCTTTTCTATGGAATAAGGAATGGACAGCAGGGGGCTGTTTCATATACTGATGACCTCTTTATAGCCAACCTTTGTTCATGGCAGCCAGCATATGGGCATATGTTGCCAAACTCTAAACCAAATACTCATTCTGATGTTTTAAATGATTTGCCCTCCCATATGTCCTTCCGAGTGAGAGACACAAAAAATTCCAACACACTATTGCAATGAAAATAAATTTCCTTTATTAGCCAGAAGTCAGATGCTCAAGGGGCTTCATGATGTCCCCATAATTTTTGGCAGAGGGAAAAAGATCTCAGTGGTATTTGTGAGCCAGGGCATTGGCCACACCAGCCACCACCTTCTGATAGGCAGCCTGCACCTGAGGAGTGAATTATTCATCATTCATCATTCCTTTTGATTCTTTGAATGCATTAAGGGGTTCCTCTACAGCTTTCCTAGGAGGTTGTATAGTCTTCTGATTGGAAGCCACAAATTTCATATAAACTAGTCCAAGTAATTGTGCTAGAGCTAAGATACTCAAACCTGAAATCAGAAGAGGGCCATGCATTGGTATACACGCCGCAATACAGAGACTCAGTCCAACCACAGCGAGGATATAGGCTATCACCTGAATAACCAATATGGCAATGACGAAGGAGGTTAATCCAATCGCTGTACTAAACACATAGTAGTGAAGTAATATTAATATCCCTGTAGAAGTCACAATTAAACCAAGGCCAGTAGCATTCTTTAATGAATATTCACCTGGGACGAAAAGAATGGCTCCAACAATGACAATGACAGTGATCACTAGTCCAGCAACAAGTAAAGCAATTGTTTTCTCATCCATACCACCGGATCTATATTTAAGTGTTTTAATACCAAACTGTCCCCAGAACAGGAGTATAGCAAAAATTGGGAAAATAACAATAAGAATATTTTCATTTGGAGAAAACCATGAAACAACACGATATTTTAGCTCGATGATCGTTTCACCTTCTCTGGTTAATTCTGTTACTTCACAAGTGTAGTTTCCTGTGTGTGAGACAGCATCACTCTTATCCATCTTCAAAGAGGCATCTCCTTTTAGTAATTGTGAGACTTCAATTTTTGCACTACTAAAGTCAGTGGGGACAGTGGACTTGTTTAGAGCTCCATCAAAGGTGTAAATATCTCTTCCTTTAAATTTCCACTTTACGTATACTTCAGTAGTGTTTTGTGCCTCCATATTAGTAACAAAGCATGGAATGACGACAGTGTCATTACAAAACGTGAATTCTACAGATTTTGTTTTATTAAATAGTAGCTGAGCTGATCCGCAGCACGCCGAGCCCAGCAACAGCGCCGCTACCAGGGGCCACATGGTGGCCAAAATGATGAGACAGCACAATAACCAGCACGTTGCCCAGGAGCTGTAGGAAAAAGAAGAAGGCATGAACATGGTTAGCAGAGGCTCTAGAGCCGCCGGTCACACGCCAGAAGCCGAACCCCGCCCTGCCCCGTCCCCCCCGAAGGCAGCCGTCCCCCCGCGGACAGCCCCGAGGCTGGAGAGGGAGAAGGGGACGGCGGCGCGGCGACGCACGAAGGCCCTCCCCGCCCATTTCCTTCCTGCCGGCGCCGCACCGCTTCGCCCCGCGCCCGCTAGAGGGGGTGCGGCGGCGCCTCCCAGATTTCGGCTCCGCACAGATTTGGGACAAAGGAAGTCCCTGCGCCCTCTCGCACGATTACCATAAAAGGCAATGGCTGCGGCTCGCCGCGCCTCGACAGCCGCCGGCGCTCCGGGGCCGCCGCGCCCCTCCCCCGAGCCCTCCCCGGCCCGAGGCGGCCCCGCCCCGCCCGGCACCCCCACCTGCCGCCACCCCCCGCCCGGCACGGCGAGCCCCGCGCCACGCCCCGCACGGAGCCCCGCACCCGAAGCCGGGCCGTGCTCAGCAACTCGGGGAGGGGGGTGCAGGGGGGGGTTACAGCCCGACCGCCGCGCCCACACCCCCTGCTCACCCCCCCACGCACACACCCCGCACGCAGCCTTTGTTCCCCTCGCAGCCCCCCCGCACCGCGGGGCACCGCCCCCGGCCGCGCTCCCCTCGCGCACACGCGGAGCGCACAAAGCCCCGCGCCGCGCCCGCAGCGCTCACAGCCGCCGGGCAGCGCGGGCCGCACGCGGCGCTCCCCACGCACACACACACGCACGCACCCCCCGAGCCGCTCCCCCCCGCACAAAGGGCCCTCCCGGAGCCCTTTAAGGCTTTCACGCAGCCACAGAAAAGAAACGAGCCGTCATTAAACCAAGCGCTAATTACAGCCCGGAGGAGAAGGGCCGTCCCGCCCGCTCACCTGTGGGAGTAACGCGGTCAGTCAGAGCCGGGGCGGGCGGCGCGAGGCGGCGCGGAGCGGGGCACGGGGCGAAGGCAACGCAGCGACTCCCGCCCGCCGCGCGCTTCGCTTTTTATAGGGCCGCCGCCGCCGCCGCCTCGCCATAAAAGGAAACTTTCGGAGCGCGCCGCTCTGATTGGCTGCCGCCGCACCTCTCCGCCTCGCCCCGCCCCGCCCCTCGCCCCGCCCCGCCCCGCCTGGCGCGCGCCCCCCCCCCCCCCGCCCCCATCGCTGCACAAAATAATTAAAAAATAAATAAATACAAAATTGGGGGTGGGGAGGGGGGGGAGATGGGGAGAGTGAAGCAGAACGTGGGGCTCACCTCGACCCATGGTAATAGCGATGACTAATACGTAGATGTACTGCCAAGTAGGAAAGTCCCATAAGGTCATGTACTGGGCATAATGCCAGGCGGGCCATTTACCGTCATTGACGTCAATAGGGGGCGTACTTGGCATATGATACACTTGATGTACTGCCAAGTGGGCAGTTTACCGTAAATAGTCCACCCATTGACGTCAATGGAAAGTCCCTATTGGCGTTACTATGGGAACATACGTCATTATTGACGTCAATGGGCGGGGGTCGTTGGGCGGTCAGCCAGGCGGGCCATTTACCGTAAGTTATGTAACGCGGAACTCCATATATGGGCTATGAACTAATGACCCCGTAATTGATTACTATTAATAACTAGTCAATAATCAATGTCGTATCGATCCTCAGCTAGCataacttcgtataaagtatcctatacgaagttat
